# Canola Root–Associated Microbiomes in the Canadian Prairies

**DOI:** 10.3389/fmicb.2018.01188

**Published:** 2018-06-08

**Authors:** Chih-Ying Lay, Terrence H. Bell, Chantal Hamel, K. Neil Harker, Ramona Mohr, Charles W. Greer, Étienne Yergeau, Marc St-Arnaud

**Affiliations:** ^1^Biodiversity Centre, Institut de Recherche en Biologie Végétale, Université de Montréal and Jardin Botanique de Montréal, Montreal, QC, Canada; ^2^Department of Plant Pathology and Environmental Microbiology, Pennsylvania State University, State College, PA, United States; ^3^Quebec Research and Development Centre, Agriculture and Agri-Food Canada, Quebec City, QC, Canada; ^4^Lacombe Research and Development Centre, Agriculture and Agri-Food Canada, Lacombe, AB, Canada; ^5^Brandon Research and Development Centre, Agriculture and Agri-Food Canada, Brandon, MB, Canada; ^6^Energy, Mining and Environment, National Research Council Canada, Montreal, QC, Canada; ^7^Centre INRS-Institut Armand-Frappier, Institut National de la Recherche Scientifique, Laval, QC, Canada

**Keywords:** rhizosphere, canola, root, *Olpidium*, PGPR, rotation, seeding density, oilseed rape

## Abstract

Canola is one of the most economically important crops in Canada, and the root and rhizosphere microbiomes of a canola plant likely impact its growth and nutrient uptake. The aim of this study was to determine whether canola has a core root microbiome (i.e., set of microbes that are consistently selected in the root environment), and whether this is distinct from the core microbiomes of other crops that are commonly grown in the Canadian Prairies, pea, and wheat. We also assessed whether selected agronomic treatments can modify the canola microbiome, and whether this was associated to enhanced yield. We used a field experiment with a randomized complete block design, which was repeated at three locations across the canola-growing zone of Canada. Roots and rhizosphere soil were harvested at the flowering stage of canola. We separately isolated total extractable DNA from plant roots and from adjacent rhizosphere soil, and constructed MiSeq amplicon libraries for each of 60 samples, targeting bacterial, and archaeal 16S rRNA genes and the fungal ITS region. We determined that the microbiome of the roots and rhizosphere of canola was consistently different from those of wheat and pea. These microbiomes comprise several putative plant-growth-promoting rhizobacteria, including *Amycolatopsis* sp., *Serratia proteamaculans, Pedobacter* sp., *Arthrobacter* sp., *Stenotrophomonas* sp*., Fusarium merismoides*, and *Fusicolla* sp., which correlated positively with canola yield. Crop species had a significant influence on bacterial and fungal assemblages, especially within the roots, while higher nutrient input or seeding density did not significantly alter the global composition of bacterial, fungal, or archaeal assemblages associated with canola roots. However, the relative abundance of *Olpidium brassicae*, a known pathogen of members of the *Brassicaceae*, was significantly reduced in the roots of canola planted at higher seeding density. Our results suggest that seeding density and plant nutrition management modified the abundance of other bacterial and fungal taxa forming the core microbiomes of canola that are expected to impact crop growth. This work helps us to understand the microbial assemblages associated with canola grown under common agronomic practices and indicates microorganisms that can potentially benefit or reduce the yield of canola.

## Introduction

Canada is one of the world's main canola-producing countries, ranking third in oil production (USDA, 2017). It is therefore important for Canada to optimize agronomic treatments in this key agro-industry. Different approaches, such as increasing seeding density, adapting fertilization regimes, and selecting optimal rotation crops and rotation sequences, were previously shown to potentially enhance canola yield or reduce disease outbreaks (Harker et al., 2003, 2012, 2015a; Guo et al., 2005; Hwang et al., 2009).

The microorganisms inhabiting plant root environments are essential in facilitating nutrient uptake, preventing colonization by pathogens, mitigating the impact of abiotic stressors, and modulating the levels of plant hormones (Yang et al., 2009; Dodd et al., 2010; Berendsen et al., 2012). Previous research revealed that canola-root-associated microbiomes are largely determined by the season and plant developmental stage (Smalla et al., 2001; Dunfield and Germida, 2003; de Campos et al., 2013). *Rhizoctonia solani* could affect the emergence and early development of canola seedlings, with impacts on the overall fungal assemblage associated with canola roots, especially in winter crops (Neupane et al., 2013a). Another study attributed a reduction in canola yield to root infection by zoospores of *Olpidium brassicae*, a known parasite of the *Brassicaceae* (Hartwright et al., 2010; Hilton et al., 2013). However, the correlations of the yields of canola and the structure of the canola root and rhizosphere microbiome have rarely been thoroughly studied. In particular, the interactions between bacterial, fungal, and archaeal communities have yet to be examined. In order to identify putatively important microorganisms in the canola root environment, we applied the concept of the core microbiome, as defined by Vandenkoornhuyse et al. (2015). In this context, the “core” microbiome of a plant species is the collection of operational taxonomic units (OTUs) that are present in any condition, while the OTUs that associate with the plant in a given condition but not under all conditions (e.g., that depend on a specific environment) are called the “eco” microbiome. Agronomic treatments that influence root-associated microbial assemblages can increase or reduce disease risks in crop production (Gomiero et al., 2011; Chaparro et al., 2012).

The aims of this work were (1) to identify and define the canola microbiome in the Canadian Prairies and to compare the bacterial, fungal, and archaeal communities associated with canola with those of reference crops; (2) to assess the effect of increased seeding density and fertilization on the canola-associated microbiomes and putative interactions between taxa from different communities; and (3) to assess the links between microbial composition and productivity metrics, including crop yield, weed counts, and seedling emergence rates. To achieve these objectives, we sampled canola roots and rhizosphere soil from an ongoing field experiment set up at three locations across western Canada (Brandon, Manitoba; Beaverlodge, Alberta; and Lacombe, Alberta). In 2014, plots were seeded with canola or with either wheat or pea as a comparison crop. The treatments applied to the canola plots included canola grown as recommended, canola fertilized at 150% of the recommended rate, and canola seeded at 150% of the recommended rate. Higher seeding density (Harker et al., 2003, 2012, 2015a,b) and higher nutrient supply (especially N) (Grant et al., 2012) were previously shown to increase canola yield in the Canadian Prairies. To our knowledge, this is the first study to comprehensively reveal the composition of all three major microbial communities of the canola microbiome using high-throughput sequencing, and to describe the core microbiome of canola. The results identify potentially important soil microorganisms for plant productivity and provide new insight into the effect of agricultural practices on canola-root-associated microbiomes.

## Materials and methods

### Description of field experiments

The present study used a subset of the plots of an experiment established in 2008 at three sites across the canola-producing area of western Canada. The sites in Lacombe (Alberta), and Brandon, (Manitoba), are in the Black soil zone, while the site in Beaverlodge (Alberta), is in the Dark Gray soil zone (Grant and Wu, 2008). At each site, the experiment is arranged in a randomized complete block design with four blocks. The initial soil characteristics are given in Table S1, and information on site management is given in Harker et al. (2015b) and Supplementary Information.

In 2014, five of the 14 treatments of the experiment were used for our study. Three of the treatments followed six years of canola monoculture, namely, (1) canola grown at 100% of the recommended rate (Can_RE), (2) canola fertilized at 150% of the recommended rate (Can_HF), and (3) canola seeded at 150% of the recommended rate (Can_HD), and the other two treatments were (4) wheat or (5) pea following canola and wheat in crop rotation systems involving canola. In 2013, the previous year, all plots had been planted with the same canola cultivar (Table 1). In the study year (2014), the plots under Can_RE were planted at the recommended seeding rate for canola, which was 100 seeds m^−2^ and received the recommended N, P, K, and S fertilization rate for canola, on the basis of soil tests. The plots under Can_HF were planted at 100 seeds m^−2^ and received 150% of the recommended fertilization rate, and the plots under Can_HD were planted at 150 seeds m^−2^ and received 100% of the recommended fertilizer rate (Table 1 and Supplementary Information). The canola cultivar was InVigor L135C (Bayer CropScience, Calgary, AB), a clubroot-resistant canola (*Brassica napus*) cultivar with resistance to the herbicide glufosinate. The wheat cultivar was Stettler, a doubled haploid hard red spring wheat (*Triticum aestivum* L.), and the pea cultivar was CDC Meadow, a yellow cotyledon field pea (*Pisum sativum* L.). Field pea and wheat were planted at the recommended rates of 100 and 300 seeds m^−2^, respectively, and were fertilized and maintained according to best management practices.

**Table 1:** Description of the crops and the treatments applied to the fields.

	**2008–2013**	**2014**					
**Treatment**	**Crop rotation^a^**	**Crop^b^**	**Seeding density (seeds m^−2^)^c^**	**Nutrient supply (%)^d^**	**Lacombe, AB**	**Brandon, MB**	**Beaverlodge, AB**
					**Actual N-P-K-S (Kg/Ha)**		
Can_RE^e^	Cr-Cr-Cr-Cr-Cr-Cr	InVigor L135C	100	100	115-46-36-0	89-34-0-10	117-25-0-0
Can_HF^e^	Cl-Cl-Cl-Cl-Cl-Cr	InVigor L135C	100	150	174-54-54-17	125-34-0-10	193-41-16-16
Can_HD^e^	Cf-Cf-Cr-Cf-Cf-Cr	InVigor L135C	150	100	115-46-36-0	89-34-0-10	117-25-0-0
Wheat	W-Cl-Cr-W-Cl-Cr	Stettler Wheat	300	100	134-46-36-0	134-51-0-14	131-25-0-0
Pea	P-W-Cr-P-W-Cr	CDC Meadow Pea	100	100	10-46-36-0	7-34-0-0	6-28-0-0

a *Cr, canola possessing the gene for resistance to glyphosate; Cl, canola possessing the gene for resistance to glufosinate herbicides; Cf, canola possessing the gene for resistance to imidazolinone*.

b *InVigor L135C is a Cl canola cultivar. The seeds were treated by Prosper Evergol, which is a combination of Clothianidin (insecticide component), Penflufen, Trifloxystrobin and Metalaxyl (3 fungicides components)*.

c *Seeding was performed with air seeders equipped with knife openers, and crops were seeded at optimal depths in 20- to 30-cm rows. Plot dimensions were 3.7 × 15.2 m*.

d *Fertilization (N, P_2_O_5_, K_2_O, and S) was based on soil tests and adjusted to achieve 100 or 150% of the recommendations for each crop species. Nitrogen was added as ESN Smart Nitrogen (Agrium Inc., Calgary, Alberta, Canada)*.

e * Canola grown as recommended (Can_RE), canola fertilized at 150% of the recommended rate (Can_HF), and canola seeded at 150% of the recommended rate (Can_HD)*.

### Sampling and additional variables

Root and rhizosphere soil samples were collected in the fourth week of July 2014, corresponding to growth stage 6 of canola (spanning from first to last flower bud opening), stage 4 of wheat development (flag leaf), and stage 6 of pea (mid-bloom) on the BBCH scale (Weber and Bleiholder, 1990). Randomly choosen plants were excavated, and fine roots with adhering soil were harvested from three or four plants at each of four random locations within each plot. In total, there was 60 plots (3 locations × 4 blocks × 5 treatments = 60 plots, including 36 canola, 12 wheat, and 12 pea plots). The roots were pooled into one composite sample per plot. In the field, immediately after collection, the root samples were placed in 15-mL Falcon tubes containing RNA*later* Stabilization Solution (Ambion, Foster City, CA, USA) for preservation. Each sample consisted of 8 mL of fine roots plus adhering soil material per plot, as measured by displacement of the RNA*later* solution. During harvest, the tubes were placed in a cooler on freezer packs. The samples were kept at 4°C before they were shipped to Swift Current, Saskatchewan, and then placed at −20°C until they were shipped by air cargo from Swift Current to Montreal, Quebec, in a Styrofoam cooler on freezer packs. Upon reception, the roots and rhizosphere soil were separated using an ethanol-sterilized sieve and scoop and were preserved at −80°C until DNA extraction. For each plot, crop emergence, crop maturity, and weed counts were recorded as described in Supplementary Information.

### DNA isolation

To isolate total genomic DNA, roots and soil samples were recovered from the RNA*later* solution and cleaned by rinsing with sterilized water. The roots were then ground using a mortar and pestle with liquid nitrogen. Total root DNA was extracted from 350 mg of root material using the NucleoSpin Soil DNA isolation kit (Macherey-Nagel, Düren, Germany) according to the manufacturer's instructions with modifications (see Supplementary Information). The final DNA yield ranged from 2 to 6 μg in 100 μL of elution solution.

Microbial genomic DNA from rhizosphere soil was isolated using the RNA PowerSoil Total RNA Isolation Kit plus the RNA PowerSoil DNA Elution Accessory Kit (Mo Bio Laboratories Inc., Carlsbad, CA, USA). The accessory kit was used because RNA had to be extracted for use in another study, and the kit allows DNA to be extracted at the same time. For each sample, 1.5–2 g of preserved rhizosphere soil was added to 2-mL microcentrifuge tubes. To reduce increases in salt content caused by the RNA*later* solution, the samples were washed in a DEPC (diethypyrocarbonate)–treated PBS (phosphate-buffered saline) solution three times before being added to the bead-beating tubes. The samples were prepared according to the manufacturer's instructions with some modifications (see Supplementary Information). The DNA products were eluted using 100 μL of elution buffer from the accessory kit and visualized on a 1% agarose gel using electrophoresis. The final DNA yield ranged from 2 to 6 μg in 100 μL of elution solution. If the appropriate band size for genomic DNA appeared, the extracts were considered usable for further analysis.

### Amplicon library preparation and sequencing

We constructed amplicon libraries for bacterial and archaeal 16S ribosomal RNA (rRNA) genes and fungal internal transcribed spacer (ITS) sequences by using target-specific PCR primers attached to Illumina overhang sequences for Nextera preparation. The primer pairs used were S-D-Bact-0341-b-S-17 with S-D-Bact-0785-a-A-21 for bacteria (Klindworth et al., 2013), ARCH517F-Illu with ARCH909R-Illu for archaea (Burggraf et al., 1997; Baker et al., 2003), and ITS1F-Illu with 58A2R-Illu for fungi (Martin and Rygiewicz, 2005; Manter and Vivanco, 2007). For each 25-μL PCR reaction of bacteria and fungi, the reaction buffer consisted of 0.5 μL each of forward and reverse primer, along with 10 μL of H_2_O, 0.5 μL of 25 mM MgCl_2_, 12.5 μL of KAPA HiFi HotStart ReadyMix (Kapa Biosystems, Cape Town, South Africa), and 1 μL of sample DNA. The 25-μL PCR reaction buffer for archaeal PCR consisted of 0.5 μL of forward and reverse primer, respectively, with 10.5 μL H2O, 12.5 μL KAPA HiFi HotStart ReadyMix, and a 1-μL sample of DNA. Reaction conditions are given in Supplementary Information.

Dual Nextera indexes were then attached to PCR products on the basis of the suggested protocol titled “16S Metagenomic Sequencing Library Preparation,” provided by Illumina, Inc. (San Diego, CA, USA), with certain modifications. The concentrated product was quantified by Qubit Fluorometric Quantitation (ThermoFisher Scientific, Waltham, MA, USA) and run on 1.5% agarose gels, and then the bands of the proper size were gel-extracted using the PureLink Quick Gel Extraction Kit (Invitrogen, Löhne, Germany) with a final elution volume of 50 μL. The gel-extraction products were quantified and then sequenced on an Illumina MiSeq sequencer using the 600-cycle MiSeq Reagent Kit v.3, according to the manufacturer's recommendations. The concentrations of samples were 33.33 nM for bacteria, 13.25 nM for archaea, and 40.00 nM for fungi. Details of the PCR reactions and indexing are available in Supplementary Information.

### Sequence trimming and bioinformatics pipelines

The preliminary processing of bacterial and archaeal 16S rRNA gene libraries was performed using mothur v.1.34.4 (Schloss et al., 2009) to join the paired ends. To reduce the file size for bacterial 16S rRNA gene sequences in order to accommodate the size limit of the 32-bit version of USEARCH (Edgar, 2010), we discarded singletons using the “unique.seqs” and “split.abund” commands with a cutoff of 1. This method replaces the equivalent procedure within USEARCH that is recommended by the Brazilian Microbiome Project (BMP) bacterial 16S pipeline (Pylro et al., 2014a,b). The database for aligning and classifying the bacterial and archaeal reads was downloaded from GreenGenes (gg_13_8_otus). Paired-end fungal reads were also joined similarly into contigs using mothur as described above, and the reads were then processed using the BMP fungal ITS pipeline (Pylro et al., 2014a,b) using QIIME (Caporaso et al., 2010), USEARCH, ITSx (Bengtsson-Palme et al., 2013), FASTA formatter (http://hannonlab.cshl.edu/fastx_toolkit/index.html), and the scripts written by the members of the BMP, with the UNITE ITS database (sh_qiime_release_s_01.08.2015) (Kõljalg et al., 2013). The identity threshold for the OTUs was set at 97%. Since the database was poorly representative of archaea, we constructed an archaeal phylogenetic tree, based on the OTUs of the members of the archaeal core microbiomes, which we had computed previously using the “compute_core_microbiome.py” script with Phylogeny.fr (Dereeper et al., 2008). The phylogenetic tree improved the identification of OTUs using reference sequences selected from GenBank. More details on bioinformatic processing are available in Supplementary Information.

The MiSeq sequencing data generated in this work were deposited in the Sequence Read Archive (SRA) database of NCBI. The NCBI Bioproject accession numbers are PRJNA383339 (Bacteria), PRJNA383353 (Fungi), and PRJNA383350 (Archaea).

### Data processing and statistical analyses

We used the QIIME script “core_diversity_analyses.py” to estimate α-diversity (Chao1, Simpson's reciprocal, and evenness indices). The effect of increased fertilization or seeding density, crop identity, and sample biotope (roots or rhizosphere soil) on the indices was tested by analysis of variance (ANOVA) with Tukey's post-hoc analyses using the “HSD.test” function of the “agricolae” package in R (https://www.r-project.org). The effect of crops and canola treatments on microbial community structure was assessed by permutational multivariate analysis of variance (PERMANOVA) using the “adonis” function of the “vegan” package in R. Two groups of comparisons were conducted: (1) the effect of crops (canola, wheat, and pea) on root and rhizosphere communities of bacteria, fungi, and archaea, and (2) the effect of canola treatments (Can_RE, Can_HF, and Can_HD) on root and rhizosphere microbial communities. To assess the clustering of samples in each group of comparisons, principal coordinates analysis (PCoA) plots were drawn using the “vegan” package in R based on the OTU matrices generated by the QIIME script “core_diversity_analyses.py” (see Supplementary Information for details).

In order to identify potentially important microorganisms in our system, we used the concept of core microbiome, as defined by Vandenkoornhuyse et al. (2015). The core microbiome associated with each crop and the eco microbiomes associated with each treatment were identified using the QIIME script “compute_core_microbiome.py”. Only the OTUs that formed more than 1% of the assemblage for at least one of the combinations of treatment and location were used. The criteria for selection and screening OTUs are detailed in Supplementary Information.

To determine whether OTUs belong to core or eco microbiomes, we compared them across crops and canola treatments, without considering location, using the QIIME script “group_significance.py” to assess their association with canola. Kruskal–Wallis tests and Benjamini–Hochberg FDR (false-discovery-rate-corrected) *P-*values (Goeman and Solari, 2014) were used to evaluate the significance of the treatment effect. When the frequency of an OTU exceeded a threshold in all samples from the three canola treatments and locations, the OTU was considered part of the canola core microbiome. If an OTU was present in one or two treatments but not in all, the OTU was considered part of the eco microbiome. The remaining OTUs constituted the unshared fraction and were not considered further. Details on the definition of the core and eco microbiomes are given in Supplementary Information. Bacterial, fungal, and archaeal OTUs of the core and eco microbiomes were analyzed across two different subgroups, (1) roots and (2) rhizospheres, and contrasted between all canola plots with reference crops (canola, wheat, and pea) and between canola treatments (Can_RE, Can_HF, and CAN_HD).

To estimate the similarity of the core microbiomes of canola, wheat, and pea, and the similarity of the core microbiomes of canola as influenced by seeding rate and fertilization, we computed the Sørensen indices using the QIIME script “beta_diversity.py” with the “binary_sorensen_dice” option. We used canola crops as the baseline for comparison when comparing the crops and Can_RE as the baseline for comparison when comparing the canola treatments. The values of the Sørensen index were tested by ANOVA with Tukey's HSD post-hoc analyses using the “HSD.test” function of the “agricolae” package in R.

The links between the bacterial and fungal core microbiomes from roots and from rhizosphere soil were separately analyzed with co-inertia analyses (CoIAs) using the “ade4” package in R. The input OTU tables were Hellinger transformed using the ‘deconstand’ function of the “vegan” package. The transformed matrices were used as input data for the principal component analysis (PCA) using the “dudi.pca” function from “ade4.” Outputs of “dudi.pca” were run with the function “coinertia” from “ade4” to generate the co-intertia analysis. The results were tested using the Monte Carlo method to obtain the simulated P value.

Redundancy analysis (RDA) was used to correlate post-spray weed counts and emergence counts with the bacterial and fungal root and rhizosphere core microbiomes and plant yields in order to assess their association with these field variables. The matrices were Hellinger-transformed using the “decostand” function followed by the “rda” function of the “vegan” package in R. The significance of the RDA model was tested by ANOVA and the *R*^2^ values were generated by the “RsquareAdj” function in R. The significance of the whole model, the axes, and each factor were also tested by ANOVA.

To identify the microorganisms correlated with yields, the association of each member of the core microbiomes of canola roots and rhizospheres with yield was tested using Spearman's correlation. The matrices were standardized using the “decostand” function of the “vegan” package in R, followed by the “rcorr” function in the “Hmisc” package. Only the members with significant correlations were included in the results.

## Results

### Taxonomic affiliations of the bacterial, fungal, and archaeal OTUs

After the reads from different data sets were filtered for quality, the bacterial gene data set allowed us to retrieve a total of 3,230,956 sequences (ranging from 3,574 to 72,331 reads across the 120 samples; Table S2) that were assigned to 6,376 OTUs after subsampling to 3,574 reads per sample. For the fungal data set, 1,112,137 sequences (ranging from 97 to 59,199 reads across 120 samples) were obtained and assigned to 679 OTUs after subsampling to 123 reads while discarding the sample with <100 reads. In the archaeal data set, we obtained a total of 2,936,314 sequences (ranging from 329 to 203,921 reads across 120 samples) that were assigned to 49 OTUs after subsampling to 329 reads per sample.

**Figure 1:**
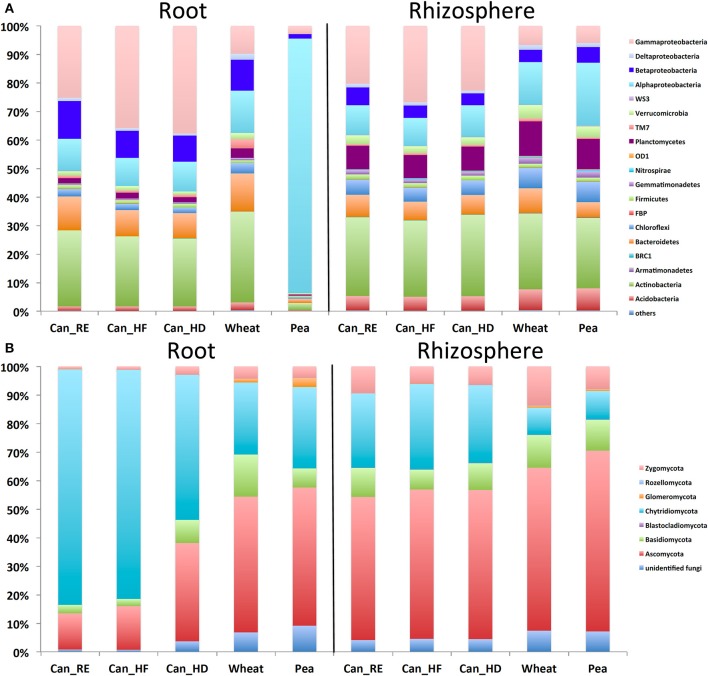
Mean (*n* = 12) relative abundances of **(A)** bacterial taxa based on the 16S ribosomal RNA (rRNA) gene fragments, and **(B)** fungal taxa based on the internal transcribed spacer (ITS) sequences in the roots and rhizospheres of canola, wheat, and pea. For canola, three different treatments were applied: canola grown as recommended (Can_RE), canola fertilized at 150% of the recommended rate (Can_HF), and canola seeded at 150% of the recommended rate (Can_HD).

Sixteen bacterial phyla with four classes from the phylum *Proteobacteria* were identified. In the canola roots (Figure 1A), the most abundant phyla/classes were *Gammaproteobacteria* (up to 40%), *Actinobacteria* (up to 50%), and *Betaproteobacteria* (up to 20%). In the canola rhizosphere, *Gammaproteobacteria* (up to 30%) and *Actinobacteria* (up to 30%) were still abundant (although lower than in the roots), followed by *Planctomycetes* (up to 15%), *Alphaproteobacteria* (up to 10%), and *Bacteroidetes* (about 5%). In comparison with canola, the reference crop pea showed a very different pattern, with *Alphaproteobacteria* forming up to 96% of bacterial taxa within the roots (Figure 1A). Wheat roots also showed a different trend in bacterial community composition at the phylum or class level, with a decrease in *Gammaproteobacteria* and an increase in *Actinobacteria* in comparison with canola. The fungal ITS data set was composed mostly of *Chytridiomycota* (up to 85% in canola roots on average) and *Ascomycota* (more in wheat or pea roots, up to 50%) (Figure 1B). *Zygomycota* were more abundant in the rhizosphere (up to 15%) than in the roots. For most of the samples, <10% of the fungal sequences were assigned to unclassified fungi. More than 99% of the archaeal reads belonged to the phylum *Thaumarchaeota*. The archaeal members that we detected in the samples were mostly unidentified, but the core members were mostly close to *Nitrocosmicus* spp. according to the phylogenetic analysis (Figure S1). Rarefaction curves showed that read abundances were close to saturation for most of the samples, and Good's coverage values ranked between 0.83 and 0.98 (Bacteria), 0.62 and 1 (Fungi), and 0.97 and 1 (Archaea) (Figure S2).

### Effect of crop identity and canola treatments on α-diversity of bacteria, fungi, and archaea

The diversity of bacterial, fungal, and archaeal assemblages was estimated using the Chao1 index, Simpson's reciprocal index, and OTU evenness, and compared between the different crops and canola treatments. For bacterial assemblages, these diversity indices were all significantly influenced by biotope (Table S3). For the archaeal community, only the Chao1 index was significantly different between biotopes. For bacteria, OTU richness was significantly higher in the rhizosphere soils than in the roots (Figure S3), irrespective of crop or treatment (Figure S3). The major differences in microbial richness were found between the root samples of the different crops, while richness was similar among the canola treatments (Figure S3). Simpson's reciprocal index and the evenness index differed between the crops in both the roots and the rhizospheres for bacteria, and the pea values were much lower than those of the other two crops (Figure S3).

For fungal assemblages, the three OTU richness indices were all significantly influenced by biotope (Table S3). In the roots, the three indices of canola significantly differed from those of wheat and pea (Figure S4). Similar trends were observed in the rhizospheres (Figure S4). However, when the three canola treatments were compared, only Simpson's reciprocal index and evenness were significantly modified in the roots (Figure S4), with higher seeding density showing higher diversity than the other treatments did. Although the biotopes had significant influence on the archaeal assemblages (Table S3), the crop types and canola treatments had no significant effect on the Chao1, Simpson's reciprocal, and evenness indices of archaeal assemblages (Figure S5).

### Effect of crops, canola treatments, and biotope on structure of microbial assemblages

**Figure 2:**
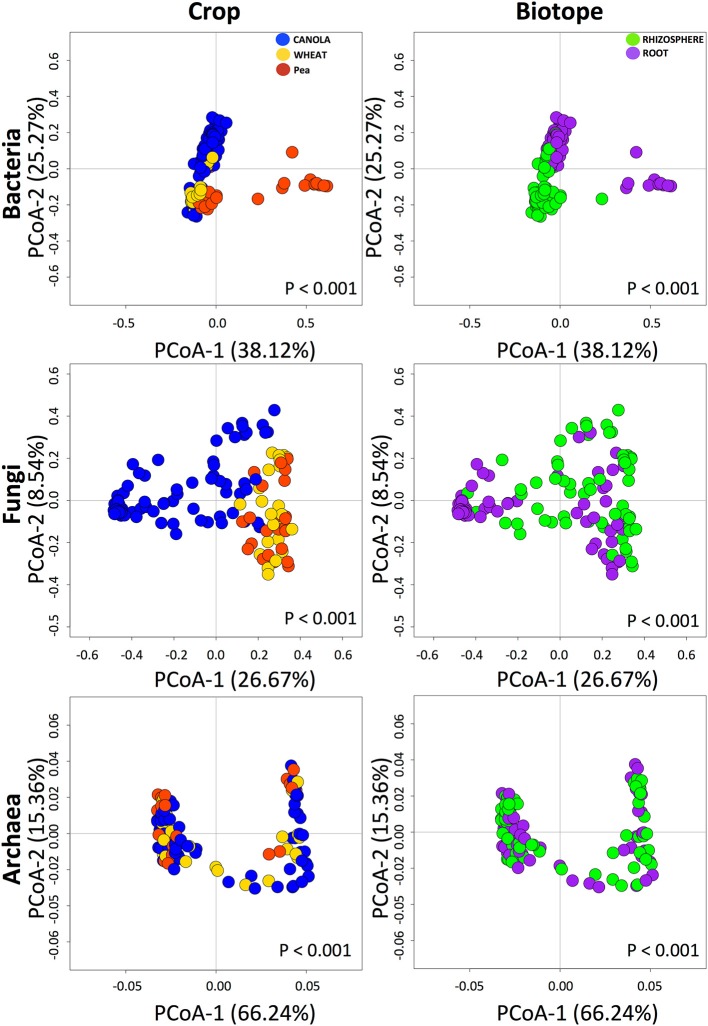
Principal coordinates analyses of the bacterial, fungal, and archaeal operational taxonomic units (OTUs), showing the grouping based on crops and biotopes. The percentages represent the variance explained by each axis. Notice that the projection range based on Bray-Curtis values of the archaeal samples was smaller than those of bacteria and fungi.

We found that bacterial, fungal, and archaeal community compositions were significantly different between crops and biotopes, with a significant interaction between crop and biotope for bacterial and fungal communities but not for archaeal communities (Table 2). The results indicate that crop type and biotope were indeed affecting microbial community composition. The pairwise comparison (Table 3) showed that bacterial communities were all significantly different either in the roots or in the rhizosphere soil between canola and the other crops. The same effects were found on the fungal community compositions (Table 3). Significant differences in archaeal community compositions occurred between canola roots and pea roots and between wheat roots and pea roots but not between canola and wheat, which shared a similar archaeal community in their roots. In the rhizosphere soils, the archaeal community composition was not different between the crops. The PCoA projections showed support for the PERMANOVA tests (Figure 2). Bacterial and fungal samples were relatively clearly grouped within crops or biotopes, especially for the pea samples, which stood out considerably from the other two crops. Since the archaeal projections are distributed in a smaller range of Bray-Curtis values, the significant effects found with PERMANOVA could not be easily visualized in the PCoA.

**Table 2:** **(A)** Effect of crop identity and biotopes on the microbial community compositions at the operational taxonomic unit (OTU) level and **(B)** effect of canola treatments and biotopes on the microbial community compositions at the OTU level, as determined by permutational multivariate analysis of variance (PERMANOVA).

	**Bacteria**			**Fungi**			**Archaea**		
	***F***	***R*^2^**	***P***	***F***	***R*^2^**	***P***	***F***	***R*^2^**	***P***
**(A) COMPARISON BETWEEN CROPS**									
Crop^a^	6.5811	0.08803	**0.001**	9.0571	0.11556	**0.001**	0.53306	0.00918	**0.001**
Biotope^b^	14.8921	0.0996	**0.001**	20.6269	0.13159	**0.001**	0.95217	0.0082	**0.001**
Crop:Biotope	3.7289	0.04988	**0.001**	2.5067	0.03198	**0.001**	0.05581	0.00096	0.975
**(B) COMPARISON AMONG CANOLA TREATMENTS**									
Treatment^c^	0.5422	0.01436	0.339	0.9714	0.02221	0.189	0.14253	0.00427	0.287
Biotope	7.7775	0.103	**0.001**	18.0785	0.20666	**0.001**	0.46114	0.00691	**0.013**
Treatment:Biotope	0.3239	0.00858	1	0.7288	0.01666	0.465	0.01297	0.00039	0.998

*Significant effects greater than or equal to P < 0.05 are indicated in bold*.

a *All canola samples (Can_RE, canola grown as recommended; Can_HF, canola fertilized at 150% of the recommended rate; and Can_HD, canola seeded at 150% of the recommended rate) were compared with wheat and pea*.

b *Roots or rhizosphere soil*.

c *Canola treatments were Can_RE, Can_HF and Can_HD*.

**Table 3:** Pairwise comparisons of the compositions of canola microbial communities with the compositions of those associated with wheat or pea, as determined by permutational multivariate analysis of variance (PERMANOVA).

**Pairwise comparisons between crops^a^**		**Bacteria**			**Fungi**			**Archaea**		
		***F***	***R*^2^**	***P***	***F***	***R*^2^**	***P***	***F***	***R*^2^**	***P***
**ROOTS**										
	Canola vs. pea	12.891	0.2189	**0.001**	8.7032	0.16206	**0.001**	0.39549	0.00852	0.04
	Canola vs. wheat	2.8499	0.05834	**0.001**	10.68	0.18843	**0.001**	0.27101	0.00586	0.136
	Pea vs. wheat	11.174	0.33683	**0.002**	2.2538	0.09692	**0.001**	0.46998	0.02092	**0.012**
**RHIZOSPHERE**										
	Canola vs. pea	1.5475	0.03255	**0.001**	3.7153	0.07473	**0.001**	0.19614	0.00425	0.253
	Canola vs. wheat	1.5296	0.03218	**0.001**	3.9191	0.07851	**0.001**	0.24029	0.0052	0.193
	Pea vs. wheat	0.96691	0.04223	**0.004**	1.493	0.06355	**0.001**	0.27558	0.01237	0.143

a *The P-value level for considering a significant difference between communities is P < 0.017 (indicated in bold when significant), following the Šidák correction for three-way comparisons. The correction was calculated using the equation 1–(1–α)^1/3^, where α is 0.05, and 3 is the number of paired comparisons*.

In a second step, the microbial community structures were compared among the different canola treatments and biotopes (Table 2). The agronomic treatments applied to the fields were not found to affect bacterial, fungal, or archaeal community composition. There was a significant difference in microbial communities between biotopes, but there was no interaction between treatment and biotope.

### Core and eco microbiomes and their variations between crops and treatments

There were 17 bacterial OTUs highly associated with canola roots (Table S4). Among those, 14 OTUs belonged to the core microbiome of canola (marked with “C” in Table S4), and were associated with the genera: *Streptomyces, Cryocola, Arthrobacter, Flavobacterium, Janthinobacterium, Serratia, Kaistobacter, Pseudomonas, Pedobacter, Agrobacterium, Burkholderia, Acidovorax, Erwinia*, and *Stenotrophomonas*. When we compared these core members of canola roots with the reference crops, we found that wheat and pea roots harbored fewer core members than canola did (wheat had 12 OTUs and pea had 1 OTU with abundance ≥ 1%; Table 4). Furthermore, wheat shared many more root core members with canola than with pea, as indicated by the lower Sørensen index of dissimilarity between canola and wheat root microbiomes (0.33) than between canola and pea root microbiomes (0.88). However, these common members still showed significant differences in abundance between crops (FDR *P*-value < 0.001; Table 4). The higher difference between canola and pea is most likely related to the presence of nodules on pea roots and to the high dominance of *Rhizobium leguminosarum* in this crop. In the rhizosphere soil, the canola bacterial core microbiome was formed of seven OTUs. Surprisingly, the bacterial core microbiome in the canola rhizosphere was more similar to the one found in the pea rhizosphere than to the one found in the wheat rhizosphere (Sørensen index = 0.34 with pea and 0.37 with wheat). It seems that the rhizosphere was a less selective environment than the roots.

**Table 4:** Comparison of the abundances of members of the canola, wheat, and pea core microbiomes.

**Most closely related taxon**		**Biotope**										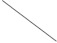
	**Root**					**Rhizosphere**						
**Phylum/class^a^**	**Genus/species/other**	**Canola^b^ (%)**	**Wheat (%)**	**Pea (%)**	**FDR *P*^c^**	**Sig^d^**	**Canola (%)**	**Wheat (%)**	**Pea (%)**	**FDR *P***	**Sig**	**OTU^f^**
Actino	*Streptomyces* sp.	**4.53**	10.63	0.66	<0.001	^***^	–^g^	–	–	–	–	OTU-B1
	*Cryocola* sp.	**2.91**	1.83	0	<0.001	^***^	**1.45**	0.86	0.51	0.32		OTU-B2
	*Arthrobacter* sp.	**2.60**	0.94	0	<0.001	^***^	**4.36**	1.41	1.25	0.001	^**^	OTU-B3
	*Blastococcus* sp.^∧e^	–	–	–	–	–	**1.17**	1.45	1.15	0.32		OTU-B6
	*Salinibacterium* sp.^∧^	0.61	1.42	0	<0.001	^***^	–	–	–	–	–	OTU-B20
	*Promicromonospora* sp.	0.46	1.08	0	<0.001	^***^	–	–	–	–	–	OTU-B21
	*Terracoccus* sp.	–	–	–	–	–	**1.02**	0.75	0.60	0.61		OTU-B5
Bact	*Flavobacterium* sp.	**3.52**	3.43	0	<0.001	^***^	–	–	–	–	–	OTU-B7
	*Pedobacter* sp.	**1.05**	1.12	0	<0.001	^***^	–	–	–	–	–	OTU-B8
α	*Kaistobacter* sp.	**1.40**	1.95	0.37	<0.001	^***^	**2.22**	3.22	2.26	0.15		OTU-B9
	*Agrobacterium* sp.	**1.14**	0.82	0	<0.001	^***^	–	–	–	–	–	OTU-B10
	*Sphingomonas* sp.	0.80	1.24	0.12	<0.001	^***^	–	–	–	–	–	OTU-B23
	*Rhizobium leguminosarum*	0.71	1.27	84.25	<0.001	^***^	0.19	0.26	9.62	<0.001	^***^	OTU-B24
β	*Janthinobacterium lividum*	**1.97**	1.27	0	<0.001	^***^	–	–	–	–	–	OTU-B11
	*Burkholderia* sp.^∧^	**1.12**	1.01	0	<0.001	^***^	–	–	–	–	–	OTU–B12
	*Acidovorax radices*^∧^	**1.60**	0.53	0	<0.001	^***^	–	–	–	–	–	OTU-B13
γ	*Serratia proteamaculans*^∧^	**9.58**	0	0	<0.001	^***^	**6.02**	0	0.17	0.001	^**^	OTU-B14
	*Pseudomonas* sp.	**5.06**	0.61	0	<0.001	^***^	**1.96**	0.41	0.74	<0.001	^**^	OTU-B15
	*Erwinia* sp.	**1.79**	0	0	<0.001	^***^	–	–	–	–	–	OTU-B16
	*Stenotrophomonas rhizophila*^∧^	**1.18**	0.28	0	<0.001	^***^	–	–	–	–	–	OTU-B18
TM7	Unknown TM7-3	0.18	1.22	0	<0.001	^***^	–	–	–	–	–	OTU-B26
Sørensen index^h^		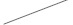	0.33 ± 0.002	0.88 ± 0.0005	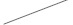	^**^	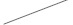	0.37 ± 0.002	0.34 ± 0.002	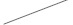	^***^	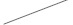
Thaumarchaeota	OTU-A1	0	0	12.31	<0.001	^***^	–	–	–	–	–	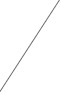
	OTU-A2	0	3.57	1.95	<0.001	^***^	0.71	0	1.65	<0.001	^***^	
	OTU-A3^i^	4.82	7.92	0	<0.001	^***^	3.39	11.73	0	<0.001	^***^	
	OTU-A4	**25.17**	25.89	18.57	0.71		**25.44**	25.15	18.34	0.65		
Sørensen index		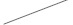	0.28 ± 0.007	0.55 ± 0.003	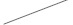	^***^	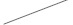	0.29 ± 0.003	0.28 ± 0.005	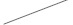		

*The bold numbers indicate members of the canola core microbiome (as shown in Table S4)*.

a *Actino, Actinobacteria; Bact, Bacteroidetes; α, Alphaproteobacteria; β, Betaproteobacteria; γ, Gammaproteobacteria; TM7, candidate phylum TM7*.

b *All canola plots included, namely, canola grown as recommended (Can_RE), canola fertilized at 150% of the recommended rate (Can_HF), and canola seeded at 150% of the recommended rate (Can_HD)*.

c *FDR P, false-discovery-rate-corrected P-value*.

d Sig, significance level:

* P < 0.05;

** P < 0.01;

*** *P < 0.001*.

e *When followed by the symbol “∧”, the classification was done by BLASTn manually with the NCBI database*.

f *OTU, operational taxonomic unit*.

g *The symbol “−” indicates that the OTU does not meet the criteria for the core/eco microbiome*.

h *The Sørensen index compares the assemblages between wheat and pea with canola*.

i *Archaeal OTU-A3 was not present in the Can_HD treatment at all, and thus we considered it to be a member of the eco microbiome of canola*.

The only archaeal member of the core microbiome of canola roots was OTU-A4 (Table S4), while OTU-A3 was found to be a member of the root eco microbiome since that OTU was not found in the Can_HD treatment (Table S4). OTU-A1 was detected only in pea roots, and OTU-A2 was detected in wheat and pea roots but not in canola roots. OTU-A4 was also present in wheat and pea roots, with similar proportions in each crop (25–26% in canola and wheat, and 19% in pea). OTU-A4 was also present in the rhizosphere of all three crops, with proportions similar to those found in roots, while OTU-A2 and OTU-A3 were detected only in canola from the Can_HF treatment. OTU-A2 was also found in the pea rhizosphere and OTU-A3 in the wheat rhizosphere. These four OTUs from the Canadian Prairies grouped closely with *Nitrocosmicus* spp. (Figure S1), including *Nitrocosmicus oleophilus* (from Korean soil samples), and *Nitrocosmicus exaquare* (from a wastewater treatment plant in Guelph, Ontario, Canada) (Sauder et al., 2017) and with the archaeal OTUs (SCA1145 and SCA1170), identified from Wisconsin agricultural soil in the United States (Bintrim et al., 1997). OTU-A2 grouped closely with SCA1170, and OTU-A1, OTU-A3, and OTU-A4 grouped with SCA1145. OTU-A4 was especially close to *Nitrocosmicus* spp., suggesting that this OTU is probably the closest to the species prevalently distributed worldwide. Also, when the substitution changes for the branches with known species were compared, the phylogenetic tree suggests that there are probably several new species of *Thaumarchaeota* to be described in the Canadian Prairies.

OTU-F9 belonging to *O. brassicae* was the only fungal OTU classified as core for canola, with abundances that varied significantly between treatments (up to 83%) in roots but not in the rhizosphere (Table S5). OTU-F9 was also detected in the microbiomes of wheat and pea roots, but in much lower abundances than in canola roots (Table S5), while it was not detected in the rhizosphere of these reference crops. In comparison with the canola fungal core microbiomes, both reference crops harbored a higher fungal diversity (Table 5). Three OTUs were shared between wheat and pea roots and were absent from canola roots. Among them, OTU-F16 was first classified as *O. brassicae* using the UNITE database (Table 5), but since OTU-F16 differed from OTU-F9 (from canola roots) (lengths of 102 vs. 132 bp; identity of 86% within 78% coverage), they were furthered compared. A BLASTn search in the NCBI database found them to be 100% identical with 100% coverage to the sequences AB205208 and AB205213, respectively classified as *O. virulentus* and *O. brassicae* in a taxonomic study of *Olpidium* spp. (Sasaya and Koganezawa, 2006) that sought to differentiate the non-virus-carrying and virus-carrying species within this genus. Our classification results were therefore updated accordingly. The OTU belonging to *O. virulentus*, which is the virus-carrying *Olpidium* species, then appeared as part of the microbiome of wheat and pea roots but was not detected in canola. Among the 15 OTUs retained as part of the fungal core microbiomes of the canola, wheat, and pea rhizospheres (Table 5), 2 OTUs were found associated with all crops, 5 OTUs were shared between canola and pea, 2 OTUs were shared between canola and wheat, 7 OTUs were shared between wheat and pea, and 5 OTUs were associated with the rhizosphere of one crop only. The Sørensen index showed that the shared fungal core and eco microbiomes were more similar between pea and canola than between wheat and canola (Table 5), either in roots or in rhizospheres.

**Table 5:** Canola core microbiomes of fungi compared with wheat and pea core microbiomes.

**Most closely related taxon**		**Biotope**										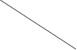
	**Root**					**Rhizosphere**						
**Taxonomy (phylum)**	**Genus/species/other**	**Canola^a^ (%)**	**Wheat (%)**	**Pea (%)**	**FDR *P*^b^**	**Sig^c^**	**Canola (%)**	**Wheat (%)**	**Pea (%)**	**FDR *P***	**Sig**	**OTU^d^**
Ascomycota	*Exophiala equina*	0	1.63	0	<0.001	^***^	0	1.42	0	<0.001	^***^	OTU-F11
	*Humicola nigrescens*	–^e^	–	–	–	–	0	2.24	0.38	<0.001	^***^	OTU-F5
	*Solicoccozyma aeria*	–	–	–	–	–	0	1.63	0.41	0.003	^**^	OTU-F6
	*Gibberella intricans*	–	–	–	–	–	0	4.34	0	<0.001	^***^	OTU-F12
	*Ilyonectria macrodidyma*	0	1.15	2.66	<0.001	^***^	0	0	2.51	<0.001	^***^	OTU-F13
	*Microdochium bolleyi*	0	6.91	0	<0.001	^***^	–	–	–	–	–	OTU-F14
	*Monographella cucumerina*	0	2.03	0	<0.001	^***^	0.68	0	3.18	<0.001	^***^	OTU-F8
	*Pseudogymnoascus roseus*	–	–	–	–	–	0	2.44	1.63	<0.001	^***^	OTU-F15
	*Nectria ramulariae*	–	–	–	–	–	0.43	0	1.15	<0.001	^***^	OTU-F4
	*Fusarium merismoides*^∧*f*^	0	0	1.85	<0.001	^***^	**7.16**	2.24	2.17	<0.001	^**^	OTU-F2
	*Fusicolla* sp.∧	–	–	–	–	–	**3.16**	1.49	1.15	0.86		OTU-F1
Chytridiomycota	*Olpidium brassicae*	**69.81**	4.67	9.16	<0.001	^***^	**26.15**	0	0	<0.001	^***^	OTU-F9
	*Olpidium virulentus*	0	13.82	18.84	<0.001	^***^	0	0	6.50	<0.001	^***^	OTU-F16
Zygomycota	*Mortierella* sp.	–	–	–	–	–	0	4.07	2.71	<0.001	^***^	OTU-F17
	*Mortierella elongata*∧	–	–	–	–	–	0	1.08	1.15	<0.001	^***^	
Unclassified	*Unidentified soil fungus*	0	2.51	4.14	<0.001	^***^	**2.33**	0	2.03	<0.001	^***^	OTU-F10
Sørensen index^g^		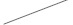	0.74 ± 0.004	0.63 ± 0.004	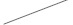		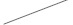	0.75 ± 0.005	0.63 ± 0.007	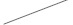		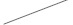

*The bold numbers indicate the members of the canola core microbiome*.

a *All canola plots included, namely, canola grown as recommended (Can_RE), canola fertilized at 150% of the recommended rate (Can_HF), and canola seeded at 150% of the recommended rate (Can_HD)*.

b *FDR P, false-discovery-rate-corrected P-value*.

c Sig, significance level:

* P < 0.05;

** P < 0.01;

*** *P < 0.001*.

d *OTU, operational taxonomic unit*.

e *The symbol “–” indicates that the OTU does not meet the criteria for the core/eco microbiome*.

f *When followed by the symbol “∧”, the classification was done by BLASTn manually with the NCBI database*.

g *The Sørensen index compares the assemblages between wheat and pea with canola*.

### Putative interactions between members of different microbial domains

**Figure 3:**
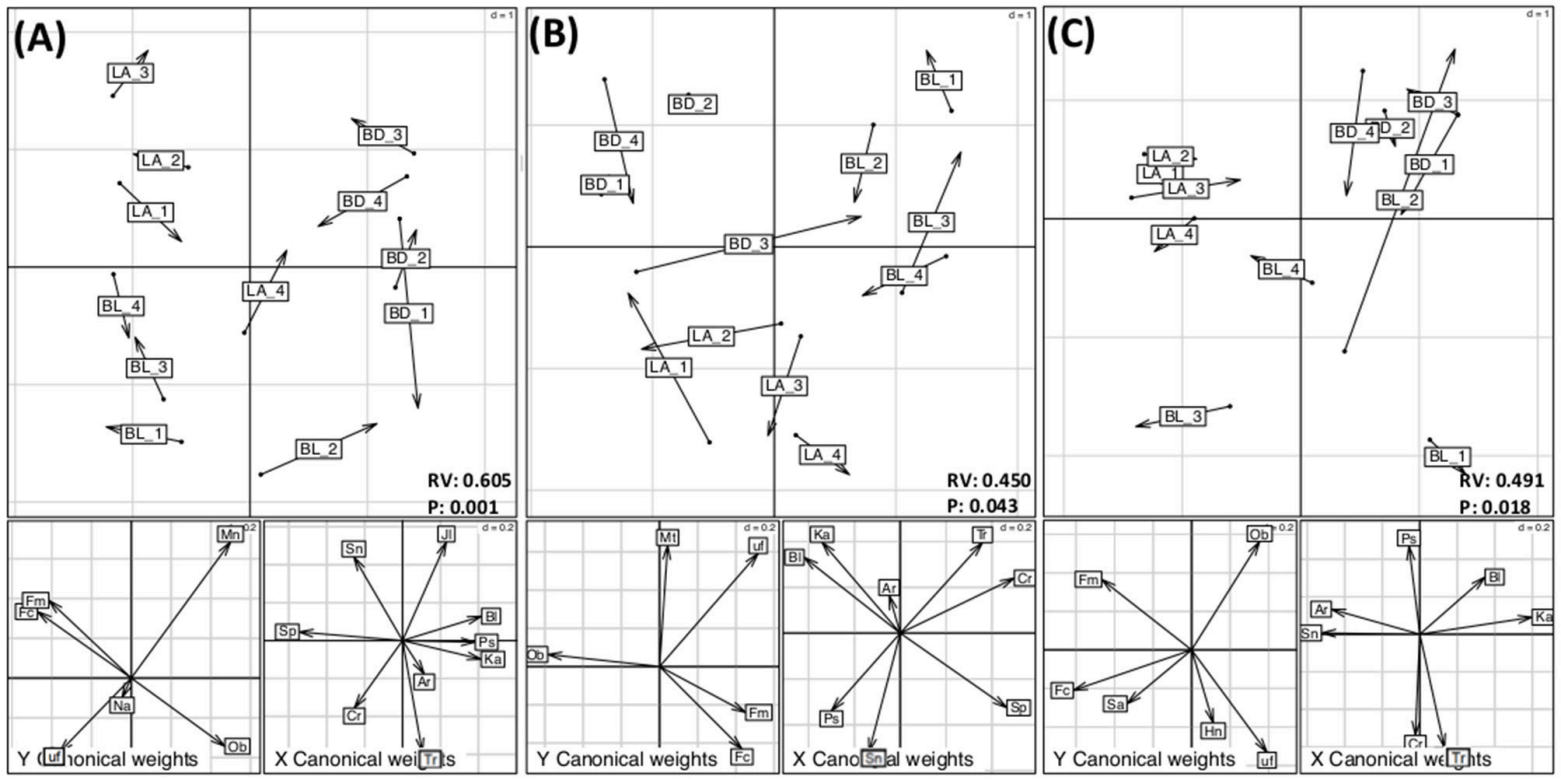
Co-inertia analysis showing the relationship between rhizosphere bacterial and fungal core and eco microbiomes in **(A)** canola grown as recommended (Can_RE), **(B)** canola fertilized at 150% of the recommended rate (Can_HF), and **(C)** canola seeded at 150% of the recommended rate (Can_HD). For each treatment, the top graph is the projection of both bacterial and fungal operational taxonomic units (OTUs) onto the co-inertia plane. The cumulative projective inertias for the first two axes were **(A)** 92.58%, **(B)** 96.49%, and **(C)** 95.88% of the total variation. The label abbreviations are as follows: BL, Beaverlodge; BD, Brandon; LA, Lacombe. Arrow length is proportional to the difference between the ordinations of the two data sets: longer arrows denote less concordance between the two assemblage data sets. The two lower panels are the projection of fungi (left panel) and bacteria (right panel) onto the co-inertia plane for each treatment. The abbreviations for bacterial and fungal species are as follows: Ar, *Arthrobacter* sp.; Bl, *Blastococcus* sp.; Cr, *Cryococcus* sp.; Jl, *Janthinobacterium lividum*; Ka, *Kaistobacter* sp.; Ps, *Pseudomonas* sp.; Sn, *Stenotrophomonas* sp.; Sp, *Serratia proteamaculans*; Tr, *Terracoccus* sp.; Fc, *Fusicolla* sp.; Fm, *Fusarium merismoides*; Hn, *Humicola nigrescens*; Ob, *Olpidium brassicae*; Mn, *Monographella cucumerina*; Mt, *Mortierella* sp.; Na, *Nectria ramulariae*; uf, unclassified fungus. The RV coefficients are the values of the multivariate generalization of the squared Pearson correlation coefficient. *P* indicates the *P*-values of each analysis.

Within each canola treatment, we examined the relationships and interactions of the bacterial and fungal core members using CoIA based on the PCA matrices, except for canola roots from the Can_HF and Can_HD treatments, where the fungal core microbiome contained only *O. brassicae*, which was not suitable to be analyzed by co-inertia analysis. This approach showed putative interactions between bacteria and fungi under different treatments and biotopes. Five main relationships were significant (*P* < 0.05), and most of them were from rhizospheres, namely, the rhizospheres of canola from the Can_RE, Can_HF, and Can_HD treatments, the rhizosphere of pea, and from the roots of wheat (Table S6, Figure 3 and Figure S6). The overall analyses showed that the bacterial and fungal core and eco microbiomes were more strongly correlated in the rhizospheres than in the roots (Table S6). The first two axes of the corresponding CoIA hyperspace (Figure 3) represented 92.58%, 96.49%, and 95.88% of the total variation for the Can_RE, Can_HF, and Can_HD treatments, respectively, in the rhizospheres. The values of the RV coefficient (a multivariate generalization of the squared Pearson correlation coefficient) for these three treatments in the rhizospheres were higher in the Can_RE treatment (0.605) than in the Can_HF (0.456) and Can_HD (0.491) treatments, indicating that the relationships between bacterial and fungal core and eco microbiomes were higher in the canola grown as recommended (Can_RE) than in the other two canola treatments. It suggests that increases in fertilization and seeding density might lower down the interactions of these two communities of microorganisms although we could not evaluate if this was positive or negative to the crops. The most obvious grouping was related to the locations. According to the projection of the fungal and bacterial OTUs on the co-inertia plane of each figure, *O. brassicae* was positively correlated with *Kaistobacter* sp. in all the canola treatments. Correlations between *O. brassicae* and *Blastococcus* sp. were stronger in the Can_HF and Can_HD treatments than in Can_RE (Figure 3A). In the Can_RE treatment, *Fusicolla* sp. and *Fusarium merismoides* were positively correlated with *Stenotrophomonas* sp. and *Serratia proteamaculans*. However, in the Can_HF plots (Figure 3B), *S. proteamaculans* was the only OTU showing a positive correlation with these two fungi. Further, in the Can_HD plots (Figure 3C), these two fungal OTUs showed a positive correlation with *Stenotrophomonas* sp. and *Arthrobacter* sp. The RV coefficient values for the pea rhizosphere and wheat root biotopes were 0.492 and 0.510, respectively. The two first axes represented 95.21 and 89.41% of the co-inertia variance (Figure S6).

### Correlation between bacterial and fungal core and eco microbiomes, and canola variables

We used RDA and Spearman's correlation to assess the relationships between the bacterial and fungal core and eco microbiomes of canola on one hand and canola yield, canola emergence, and post-spray weed count (Figure S7) on the other hand. Overall, location was the only factor that was found to significantly affect all members of the bacteria and fungi core and eco microbiomes as well as canola yield, according to the RDA model. However, neither post-spray weed counts nor emergence counts were significantly correlated with the core and eco microbiome members. Spearman's correlation showed that individual members of the core and eco microbiomes (Table 6) that were significantly correlated with yield were mostly bacteria and were mostly members of the core microbiomes, either of the roots or of the rhizosphere. Three fungal members of the rhizosphere microbiome were found to be significantly correlated with yield: *Monographella cucumerina* (negatively correlated), *Fusicolla* sp. (positively correlated), and *F. merismoides* (positively correlated). In roots, five bacteria were positively correlated with yield: *Amycolatopsis* sp., *Stenotrophomonas* sp., *S. proteamaculans, Arthrobacter* sp., and *Pedobacter* sp. The remaining bacteria were negatively correlated with yield. *Amycolatopsis* sp. and *Stenotrophomonas* sp. were members of the eco microbiome associated with higher fertilization (Can_HF treatment). In the rhizosphere, *S. proteamaculans* and *Stenotrophomonas* sp. were also positively correlated with yield. *S. proteamaculans* was also a member of the eco microbiome associated with higher fertilization. Interestingly, in the rhizosphere, *Fusicolla* sp. and *F. merismoides*, which can be considered potential pathogens (Gräfenhan et al., 2011), showed positive correlations with yield. On the other hand, the very abundant *O. brassicae* was not significantly correlated with canola yield. The canola archaeal core OTUs did not show significant correlation with canola yield.

**Table 6:** Bacterial and fungal taxa from the core and eco microbiomes of canola showing a significant Spearman's correlation between their relative abundance and canola yield.

**Biotope**		**Name^a^**	***P***	**Rho**	**Microbiome^b^^,^^c^**
*Bacteria*	*Root*	*Enterobacter* sp.	0.0445	−0.337	Eco (Can_HF)
		***Amycolatopsis*** **sp**.	**0.00213**	**0.495**	**Eco (Can_RE, Can_HF)**
		***Stenotrophomonas*** **sp**.	**0.00702**	**0.441**	**Eco (Can_HF, Can_HD)**
		*Burkholderia* sp.	0.00667	−0.444	Core
		*Flavobacterium* sp.	0.0115	−0.417	Core
		*Janthinobacterium lividum*	0.00468	−0.461	Core
		*Pseudomonas* sp.	0.00468	−0.461	Core
		***Serratia proteamaculans***	<**0.001**	**0.680**	**Core**
		***Arthrobacter*** **sp**.	<**0.001**	**0.710**	**Core**
		***Pedobacter*** **sp**.	**0.00590**	**0.450**	**Core**
		*Agrobacterium* sp.	<0.001	−0.583	Core
		*Flavobacterium* sp.	0.0115	−0.417	Core
		*Janthinobacterium lividum*	0.00468	−0.461	Core
		*Acidovorax radices*	0.00411	−0.467	Core
	*Rhizosphere*	*Janthinobacterium lividum*	0.00795	−0.435	Eco (Can_RE)
		***Serratia proteamaculans***	<**0.001**	**0.727**	**Eco (Can_RE, Can_HF)**
		*Blastococcus* sp.	<0.001	−0.695	Core
		***Stenotrophomonas*** **sp**.	**0.0114**	**0.417**	**Core**
		*Kaistobacter* sp.	<0.001	−0.750	Core
*Fungi*	*Rhizosphere*	*Monographella cucumerina*	0.00856	−0.432	Eco (Can_RE)
		***Fusicolla*** **sp**.	<**0.001**	**0.559**	**Eco (Can_RE, Can_HF)**
		***Fusarium merismoides***	**0.0213**	**0.383**	**Core**

a *Positive relationships are indicated in bold*.

b *No significant correlations were found between yield and root-associated fungal taxa*.

c *Can_RE, canola grown as recommended; Can_HF, canola fertilized at 150% of the recommended rate; Can_HD, canola seeded at 150% of the recommended rate*.

## Discussion

In this study, we compared the canola root-associated microbiome with those of wheat and pea grown alongside canola in the same fields, and the effect of selected agronomic treatments on the canola microbiome. We assessed the effect of crop and treatment on the α- and β-diversities of the bacterial, fungal, and archaeal assemblages associated with the roots and rhizosphere soil. The results show that canola has a core microbiome distinct from those of wheat and pea and that the root and rhizosphere microbiomes significantly responded to the agronomic treatments. We also found treatment-specific changes in the relationship between bacterial and fungal microbiome members.

### Canola microbiome

The canola microbiome was significantly different from those of the reference crops in all three microbial domains, both in the roots and in the rhizosphere soil, according to PERMANOVA and core microbiome analyses. Different plant species have often been reported to select for different root-associated microorganisms (e.g., Hallmann et al., 1997; Tkacz et al., 2015). Previous work has been done on the canola-root-associated bacterial community (Germida et al., 1998; Macrae et al., 2000; Alström, 2001; Misko and Germida, 2002; Farina et al., 2012; Croes et al., 2013; de Campos et al., 2013). Table S7 provides a list of the bacterial taxa forming the core microbiomes of canola roots and rhizosphere from the present study in comparison with previous research and the PGPR references (Kloepper et al., 1988; Belimov and Dietz, 2000; Alström, 2001; Poly et al., 2001; Gray and Smith, 2005; Cruz et al., 2008; Kumar et al., 2011; Pliego et al., 2011). Although the studied areas were quite diverse, there were a large number of common species found in each study. In our study, the core microbiome encompasses 12 different genera that were also detected in previous research. In particular, *Pseudomonas* spp. and *Stenotrophomonas* sp. were detected in five previous studies (Table S7). According to Table 4, these two bacterial genera were present at very low abundance or were absent from the wheat and pea core microbiomes. This observation indicates that these taxa are quite unique to the canola microbiome. Interestingly, the members of the canola core microbiome that we identified were not that similar to those in the other two Canadian studies by Misko and Germida (2002) and Germida et al. (1998). The difference was probably due to different sampling times and locations since the microbiome was shown to change significantly between different growth stages (Farina et al., 2012) and soil types (Garbeva et al., 2004).

The three agronomic treatments applied to the canola plots consisted of the recommended practice as well as a higher fertilization rate and a higher seeding density. Higher fertilization rate and seeding density were shown to increase canola yields (as detailed in the introduction). In previous studies, higher fertilization of wheat fields also increase soil microbial biomass and activities, as determined by enzyme assays (Goyal et al., 1999; Raiesi, 2004). Our results show that the abundances of *Amycolatopsis* sp., *Enterobacter* sp., *Stenotrophomonas* sp., *Janthinobacterium lividum, S. proteamaculans*, and the archaeal OTU-A2 and OTU-A3 were significantly affected by the treatments in canola roots or rhizosphere. The other OTUs remained quite stable between the different treatments. Except for *Enterobacter* sp., *J. lividum*, and the archaeal OTUs, these microbial taxa were all positively correlated with canola yield. In addition, two members of the core microbiome that were not affected by the treatments, *Arthrobacter* sp. and *Pedobacter* sp., were also positively correlated with yield.

Even with a relatively low Good's coverage (0.62) for fungi, due to subsampling to 123 reads per sample, the analysis showed that the situation was quite different with the canola fungal core microbiome. In previous studies (Bennett et al., 2014; Tkacz et al., 2015; Gkarmiri et al., 2017), *O. brassicae*, a fungal parasite, was found to be the dominant and most active fungal species in the canola rhizosphere and root environments. Our results confirmed these findings; moreover, in our study, it was the only fungus forming the core microbiome of canola roots. However, the abundance of this taxon was highly reduced in wheat and pea roots, along with a concurrent increase in the abundance of *O. virulentus*, which was the dominant fungus in the roots of those crops. In canola roots, the abundance of *O. brassicae* was also highly reduced in densely seeded plots in comparison with the other agronomic treatments, perhaps because of the higher soil-drying potential of a dense plant stand, or due to the higher amount of fungicide brought to the same volume of soil since the commercial seeds were coated with pesticides. Nevertheless, in our study, the abundance of *O. brassicae* was not negatively correlated with canola yield. A previous study (Hilton et al., 2013) showed that infection with a high number of *O. brassicae* zoospores reduced the above-ground growth and root growth of canola seedlings and also affected pod and seed production. The absence of a negative correlation with yield in the present study suggests that in our experiment, either the minimum threshold of *Olpidium* zoospores at the sensitive growth stage was not met or the environmental conditions that are required for disease expression were not present. Further research would therefore be needed to determine if increasing the seeding density of canola would reduce the detrimental effect of *O. brassicae* when the conditions required for disease development are present. We also observed a positive statistical interaction between the OTUs of *O. brassicae* and those of *Kaistobacter*, based on Co-intertia analysis. These two taxa were both recently found among the most active microbes in the rhizosphere of *B. napus* (Gkarmiri et al., 2017). While the role of *Kaistobacter* in canola rhizosphere is unknown, more investigation would be required to determine if the biological interaction between these species is direct or indirect.

### Potential plant-growth-promoting microorganisms and their putative effects

Many microorganisms promote plant growth (Gray and Smith, 2005), but evidence shows that some species of microorganisms acting as plant-growth-promoting microorganisms in most conditions can also reduce crop yield under certain circumstances (Bennett et al., 2012). Thus, while identifying PGPRs, one needs to consider the conditions where the PGPR were observed. In this study, we assessed the members of the core and eco microbiomes of canola using Spearman's correlation analysis to identify the microorganisms related to canola yield, in order to highlight their potentially beneficial or detrimental effect on canola productivity. It is well known that plant growth promoting characteristics are strain-dependent so these correlations are only indicative of potential PGPR microorganisms. Sixteen bacterial OTUs and three fungal OTUs, mainly from the root microbiomes, were significantly correlated with canola yield. Among them, OTUs related to *Amycolatopsis* sp., *S. proteamaculans, Pedobacter* sp., *Arthrobacter* sp., *Stenotrophomonas* sp., *F. merismoides*, and *Fusicolla* sp. were positively related to yield (Table 6), while others, including some supposed PGPR such as *Flavobacterium* sp. and *Pseudomonas* sp., were negatively correlated with canola yield.

*Serratia proteamaculans* is well known as a PGPR that facilitates nodulation and nitrogen fixation in soybeans and lentils (Chanway et al., 1989; Dashti et al., 1998). This taxon was also shown to promote growth and resistance to fungal pathogens in rapeseed (Alström, 2001; Neupane et al., 2013b). As a member of the canola root core microbiome in our work, this species had high specificity to canola, but in contrast, pea and wheat did not appear to recruit the OTU related to *S. proteamaculans* as a member of their core microbiomes. In our study, the OTU related to *S. proteamaculans* is also a potentially beneficial rhizosphere and root microorganism that was highly correlated with canola yield.

*Arthrobacter* spp. has been studied in fields for a long time and was shown in a previous study to increase canola yield significantly when applied as a bacterial suspension to seeds (Kloepper et al., 1988). *Arthrobacter* was also recognized as fast-growing bacteria in the rhizosphere of *B. rapa* canola (Tkacz et al., 2015). In our study, one OTU related to *Arthrobacter* was a member of the canola root core microbiome, with significantly lower abundances in pea and wheat. The fact that *Arthrobacter* had the highest correlation with canola yield among the members of the root core microbiome suggests an important beneficial influence of this bacterium on canola.

The OTU related to an unknown *Stenotrophomonas* sp. present in canola roots and rhizosphere was significantly correlated with yield. The related bacterium *Stenotrophomonas rhizophila*, which was originally identified from oilseed rape and potato roots and was reported to have antagonistic activity against fungal plant pathogens (Wolf et al., 2002), was also present in the core microbiome of canola. *Stenotrophomonas* sp. was previously shown to inhibit the growth of *Verticillium dahliae* in oilseed rape plants (Alström, 2001). The OTU related to unknown *Stenotrophomonas* was more abundant in the canola plots in the high-seeding-rate and high-fertilization-rate treatments, while *S. rhizophila* seemingly benefits from the use of a high fertilization rate. Therefore, it is unclear whether those species are beneficial to canola or whether they instead benefit from large, well-fertilized canola crops.

Endophytism was reported in several *Amycolatopsis* spp. (Miao et al., 2011; Xing et al., 2013; Klykleung et al., 2015; Axenov-Gribanov et al., 2016), and accordingly, an OTU related to *Amycolatopsis* sp. was detected only in the roots of canola in our study. Some *Amycolatopsis* spp. have antimicrobial and antifungal activities (Saito et al., 2009; Axenov-Gribanov et al., 2016), which may explain the positive correlation of this bacterium with canola yield in our experiment. Another OTU related to a commonly observed endophyte, *Pedobacter* sp., was detected in the roots of canola and wheat in our experiment. This OTU was positively correlated with canola yield, which may be explained by the bacterium's ability to produce indole acetic acid (IAA) (Yuan et al., 2011). *Pedobacter* sp. was reported in a previous study to be more abundant at the flowering stage of canola (de Campos et al., 2013). The production of IAA by this bacterium may trigger the flowering of canola, which could explain the presence of a positive correlation with yield.

*Fusarium* spp. and *Fusicolla* spp. are closely related (Gräfenhan et al., 2011), and many species from these two genera are plant pathogens (van der lee et al., 2015). Two OTU detected in the canola rhizosphere showed best match to sequences from these genera and were positively related to canola yield. However, it should be noted that the short ITS barcode sequences used in this study are known to not be particularly suitable for *Fusarium* species identification. Thus, these identifications should be interpreted with caution and further work will be required to confirm their identity more accurately. The *Fusarium* OTU that we detected in the rhizosphere was related to *F. merismoides*, a species considered to cause tomato stem rot (Fletcher and Lord, 1985). Non-pathogenic *Fusarium* spp. were sometimes detected in healthy roots from *Fusarium*-suppressive soils, and these non-pathogenic species or strains may antagonize pathogenic *Fusarium* spp. through competition. However, the cause of the suppression may also be the presence of other microorganisms in the soils (Weller et al., 2002), such as certain *Actinomycetes* and *Gammaproteobacteria*, including some species of pseudomonads (Haas and Défago, 2005). Another study showed that indigenous bacterial species, including *Stenotrophomonas* spp. and *Serratia* spp., could parasitize pathogenic *Fusarium*, reducing the production of aerial hyphae and microconidia (Minerdi et al., 2008). The colonization of *Fusarium* by beneficial bacteria may explain the positive correlations found between *Fusarium* spp. and *Fusicolla* spp. on one hand and canola yield on the other hand. The CoIA clearly showed a correlation between the two potentially pathogenic fungal species, *F. merismoides* and *Fusicolla* sp., and two potentially beneficial bacteria, *Stenotrophomonas* sp. and *S. proteamaculans*. Previous studies reported that the incidence of Fusarium disease and *Fusarium* populations was related to changes in soil microbial community structure in asparagus (Hamel et al., 2005; Yergeau et al., 2010). It was also shown that there is no relationship between the abundance of *Fusarium* in soil and Fusarium disease, and even no clear relationship between the presence of *Fusarium* in plant roots and Fusarium disease (Vujanovic et al., 2006; Yergeau et al., 2006).

Although some members of *Pseudomonas* and *Flavobacterium* are generally reported to be beneficial bacteria in most studies (Poly et al., 2001; Gray and Smith, 2005; Farina et al., 2012; de Campos et al., 2013), some records indicate that *Pseudomonas* spp. and *Flavobacterium* spp. may produce chemicals inhibiting plant growth (Bennett et al., 2012). Even though Pseudomonas is a broad genus, the negative relationships of the OTUs related to this genus with plant yield that we found concur with these reports.

### Archaeal assemblage and canola

Archaea have traditionally been considered to be extremophiles, but mesophilic archaea were found in the soil of soybean and paddy fields (Ueda et al., 1995; Kudo et al., 1997). Archaea were also observed on the rhizoplane and older root hairs of tomato by means of *in situ* staining and microscopy (Simon et al., 2000). The richness of archaeal assemblages was found to be higher in plant rhizospheres than in bulk soils of 76 different plant samples from diverse locations but plant identity had no influence (Sliwinski and Goodman, 2004). Archaea related to the genus *Nitrososphaera* were recently detected in sorghum and sunflower rhizospheres (Oberholster et al., 2018). Mesophilic archaea are also well-known as ubiquitous ammonia oxidizers (Kim et al., 2011). In our study, most archaeal OTUs belonged to Thaumarchaeota, and a similar non-specific prevalence happened only with OTU-A4, which was similarly abundant in all crops and under all agronomic treatments. However, PERMANOVA tests indicated that the relative abundance in the overall archaeal community and the archaeal OTUs forming the core microbiome significantly changed between crops. Contrary to the active components of *B. napus* roots/rhizosphere archaea that were reported to be mostly related to *Nitrososphaera* (Gkarmiri et al., 2017), all of the core archaeal OTUs we found here were closer to *Nitrocosmicus* spp. The abundance of OTU-A2 and OTU-A3 in canola roots and rhizosphere soil also shifted significantly with treatments, especially with the addition of fertilizers. Although we did not detect any correlation between the members of the canola archaeal core microbiome and canola yield, the significant differences in abundance of OTU-A2 and OTU-A3 in the high-fertilization-rate treatment suggest that the members of *Thaumarchaeota* may benefit from the organic matter released from plant roots or utilize ammonia in the root environment. However, the way in which canola plants may influence and manipulate *Archaea* is still unknown.

## Conclusion

In this work, we described canola microbiomes for three communities of microorganisms, namely, bacteria, fungi, and archaea. We found that canola microbiomes were distinguished between the two biotopes (roots and rhizosphere) and were significantly different from those of the reference crops (wheat and pea). We highlighted the potential PGPR among those microorganisms by correlating the core microbiome members in the Canadian Prairies with canola yield. Taxa related to *Amycolatopsis* sp., *S. proteamaculans, Pedobacter* sp., *Arthrobacter* sp., *Stenotrophomonas* sp., *F. merismoides*, and *Fusicolla* sp. are potentially beneficial to canola due to their status as members of the core or eco microbiome and their positive correlation with canola yield. Fertilization and seeding rates seem to influence certain taxa forming the core and eco microbiomes of canola based on the relative abundances profiles, notably the parasite *O. brassicae* which was less abundant at the higher seeding rate. Certain archaeal taxa showed some specificity to crops and treatments. Furthermore, the putative interactions between the members of bacterial and fungal core microbiomes were weaker with higher fertilization and seeding than the recommended treatments in canola rhizospheres. Our study provides information about the canola root microbiome that is fundamental for the design of microbiome management strategies for improving canola yield and health.

## Author contributions

KH, TB, CH, and MS-A conceived and designed the experiments. KH, RM, and C-YL performed the experiments. C-YL analyzed the data. MS-A, CG, and EY contributed reagents, materials, analysis tools. C-YL, MS-A, CH, TB, and EY wrote the paper.

### Conflict of interest statement

This research was supported by Agri-Food Canada and the Canola Council of Canada, but these partners have in no way influenced or modified this manuscript or the analysis of the results presented. The authors declare that the research was conducted in the absence of any commercial or financial relationships that could be construed as a potential conflict of interest.

## Supplementary material

Figure S1 Maximum likelihood phylogenetic tree based on 175 bases of the archaeal 16S ribosomal RNA (rRNA) fragments. The branch length is proportional to the number of substitutions per site.

Figure S2 Rarefaction curves based on Good's coverage per sample of **(A)** bacterial 16S rRNA, **(B)** fungal internal transcribed spacer (ITS), and **(C)** archaeal 16S rRNA libraries.

Figure S3 Biodiversity indices [Chao1, Simpson's reciprocal (Simpson R), and evenness] of bacterial assemblages based on the operational taxonomic units (OTUs) of 16S rRNA gene fragments. The figure shows the comparisons between crops **(left)** and canola treatments **(right)**. The canola treatments were canola grown as recommended (Can_RE), canola fertilized at 150% of the recommended rate (Can_HF), and canola seeded at 150% of the recommended rate (Can_HD). The black and white boxes represent the median to the upper quartile and the median to the lower quartile, respectively.

Figure S4 Biodiversity indices [Chao1, Simpson's reciprocal (Simpson R), and evenness] of fungal assemblages based on the operational taxonomic units (OTUs) of internal transcribed spacer (ITS) fragments. The figure shows the comparisons between crops **(left)** and canola treatments **(right)**. The canola treatments were canola grown as recommended (Can_RE), canola fertilized at 150% of the recommended rate (Can_HF), and canola seeded at 150% of the recommended rate (Can_HD). The black and white boxes represent the median to the upper quartile and the median to the lower quartile, respectively.

Figure S5 Biodiversity indices [Chao1, Simpson's reciprocal (Simpson R), and evenness] of archaeal assemblages based on the operational taxonomic units (OTUs) of 16S rRNA fragments. The figure shows the comparisons between crops **(left)** and canola treatments **(right)**. The canola treatments were canola grown as recommended (Can_RE), canola fertilized at 150% of the recommended rate (Can_HF), and canola seeded at 150% of the recommended rate (Can_HD). The black and white boxes represent the median to the upper quartile and the median to the lower quartile, respectively.

Figure S6 Co-inertia analysis showing the relationship between bacterial and fungal core and eco microbiomes in **(A)** the rhizosphere of pea and **(B)** the roots of wheat. For each crop, the top graph is the projection of both bacterial and fungal operational taxonomic units (OTUs) onto the co-inertia plane. The cumulative projective inertias for the first two axes were **(A)** 95.21% and **(B)** 89.41% of the total variation. The label abbreviations are as follows: BL, Beaverlodge; BD, Brandon; LA, Lacombe. Arrow length is proportional to the difference between the ordinations of the two data sets: longer arrows denote less concordance between the two assemblage data sets. The two sub-panels are the projection of fungi (left sub-panel) and bacteria (right sub-panel) onto the co-inertia plane for each crop. The abbreviations for bacterial and fungal species are as follows: Ac, *Acidovorax radius*; Ar, *Arthrobacter* sp.; Ag, *Agrobacterium* sp.; Bl, *Blastococcus* sp.; Bk, *Burkholderia* sp.; Cr, *Cryococcus* sp.; Fl, *Flavobacterium* sp.; Jl, *Janthinobacterium lividum*; Ka, *Kaistobacter* sp.; Pe, *Pedobacter* sp.; Pm, *Promicromonospora* sp.; Ps, *Pseudomonas* sp.; Rl, *Rhizobium leguminosarum*; Sg, *Sphingomonas* sp.; Sl, *Salinibacterium* sp.; Sn, *Stenotrophomonas* sp.; Sp, *Serratia proteamaculans*; St, *Streptomyces* sp.; Tm, TM7-3; Tr, *Terracoccus* sp.; Ex, *Exophiala equine*; Hn, *Humicola nigrescens*; Im, *Ilyonectria macrodidyma*; Ob, *Olpidium brassicae*; Ov, *Olpidium virulentus*; Mb, *Microdochium bolleyi*; Mn, *Monographella cucumerina*; Mt, *Mortierella* sp.; Na, *Nectria ramulariae*; Pr, *Pseudogymnoascus roseus*; uf, unclassified fungus. The RV coefficients are the values of the multivariate generalization of the squared Pearson correlation coefficient. *P* indicates the *P*-values of each analysis.

Figure S7 Agronomic variables of **(A)** canola emergence counts, **(B)** post-spray weed count, and **(C)** canola yield. The canola treatments were canola grown as recommended (Can_RE), canola fertilized at 150% of the recommended rate (Can_HF), and canola seeded at 150% of the recommended rate (Can_HD).

Table S1 Soil properties at each location.

Table S2 The raw sequencing counts of each sample for bacteria, fungi and archaea by MiSeq Sequencing.

Table S3 Effects of crop identity and canola treatments on the Chao1, Simpson's reciprocal (Simpson R), and evenness indices, as determined by analysis of variance (ANOVA). Significant effects greater than *P* < 0.05 are indicated in bold.

Table S4 Canola bacterial and archaeal core and eco microbiomes among different treatments.

Table S5 Canola fungal core and eco microbiomes among different treatments.

Table S6 Significance of the relationships between the bacterial and fungal core/eco microbiomes, as determined by co-inertia analysis.

Table S7 Comparison of the bacterial core microbiome of canola from this study with bacterial taxa considered important in other studies.
