# Supplementary figures and images for: Canola Root–Associated Microbiomes in the Canadian Prairies
Source: Front Microbiol. 2018 Jun 8;9:1188. doi: 10.3389/fmicb.2018.01188 (PMC6002653; doi:10.3389/fmicb.2018.01188)

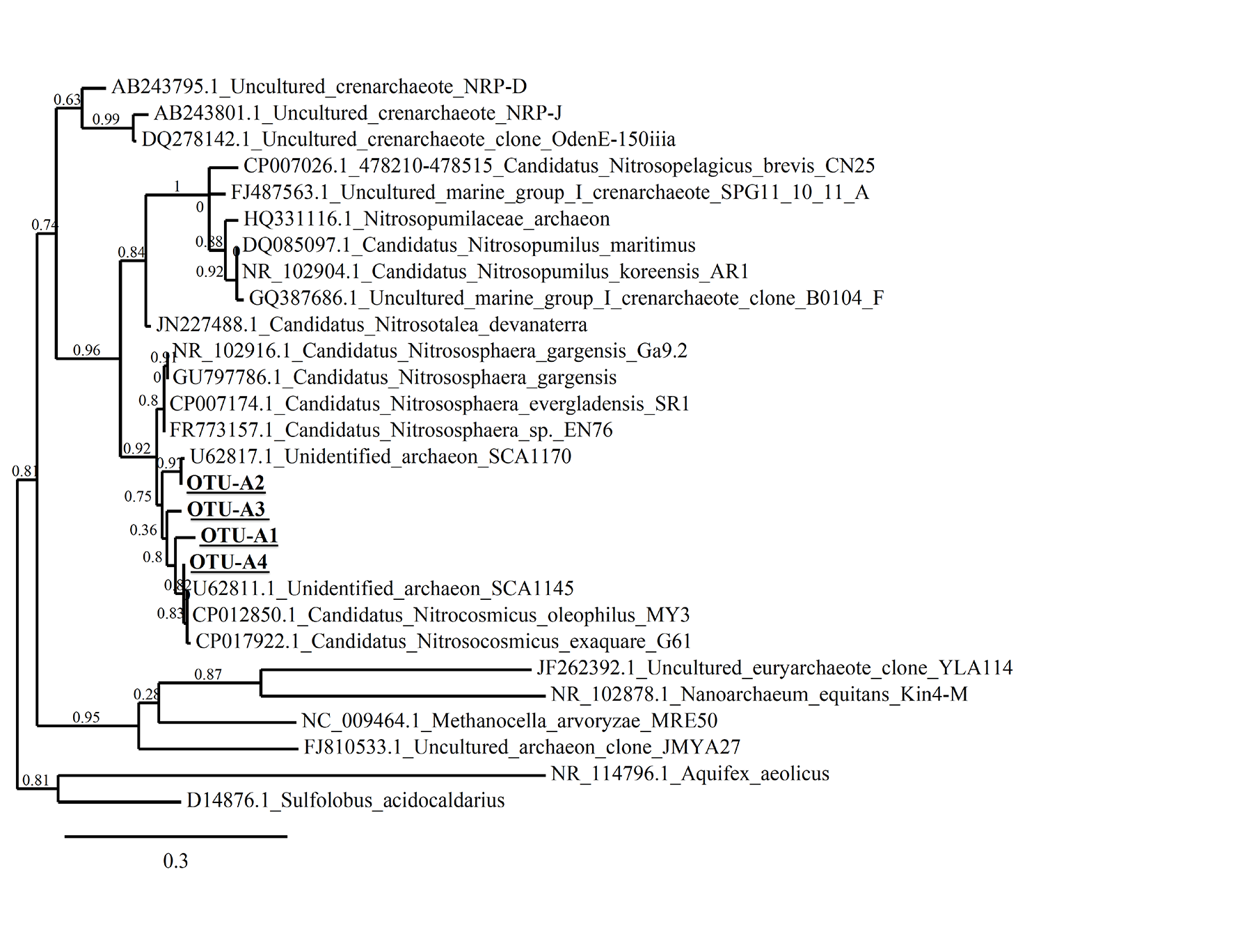

Supplement: Figure S1 — Maximum likelihood phylogenetic tree based on 175 bases of the archaeal 16S ribosomal RNA (rRNA) fragments. The branch length is proportional to the number of substitutions per site. [file Image_1.TIFF]

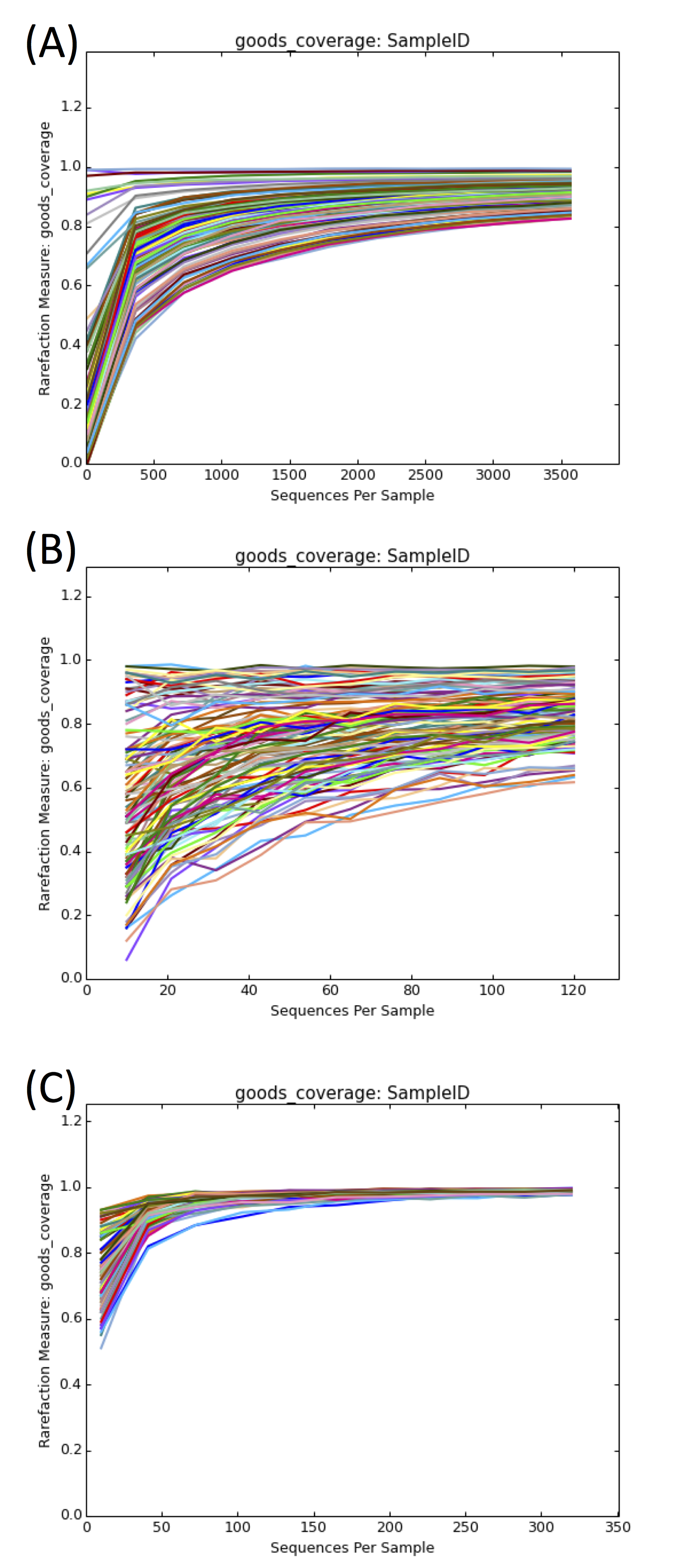

Supplement: Figure S2 — Rarefaction curves based on Good's coverage per sample of (A) bacterial 16S rRNA, (B) fungal internal transcribed spacer (ITS), and (C) archaeal 16S rRNA libraries. [file Image_2.TIFF]

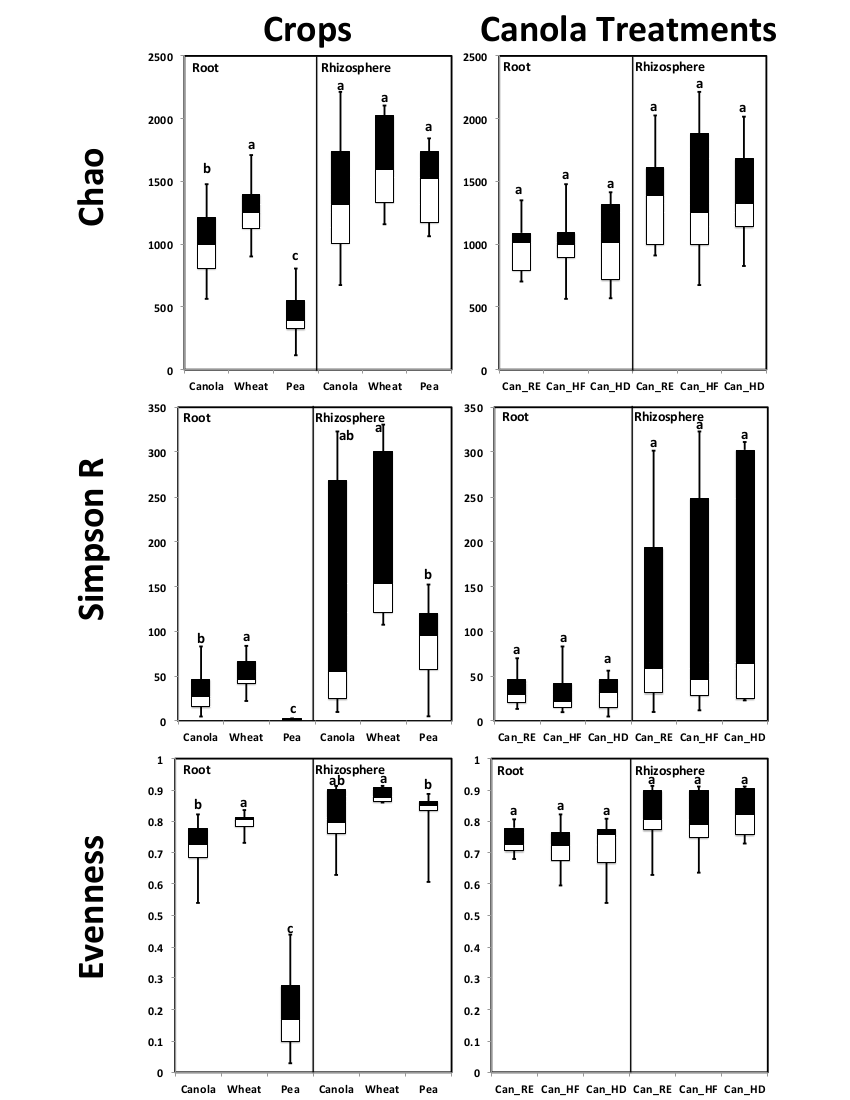

Supplement: Figure S3 — Biodiversity indices [Chao1, Simpson's reciprocal (Simpson R), and evenness] of bacterial assemblages based on the operational taxonomic units (OTUs) of 16S rRNA gene fragments. The figure shows the comparisons between crops (left) and canola treatments (right). The canola treatments were canola grown as recommended (Can_RE), canola fertilized at 150% of the recommended rate (Can_HF), and canola seeded at 150% of the recommended rate (Can_HD). The black and white boxes represent the median to the upper quartile and the median to the lower quartile, respectively. [file Image_3.TIFF]

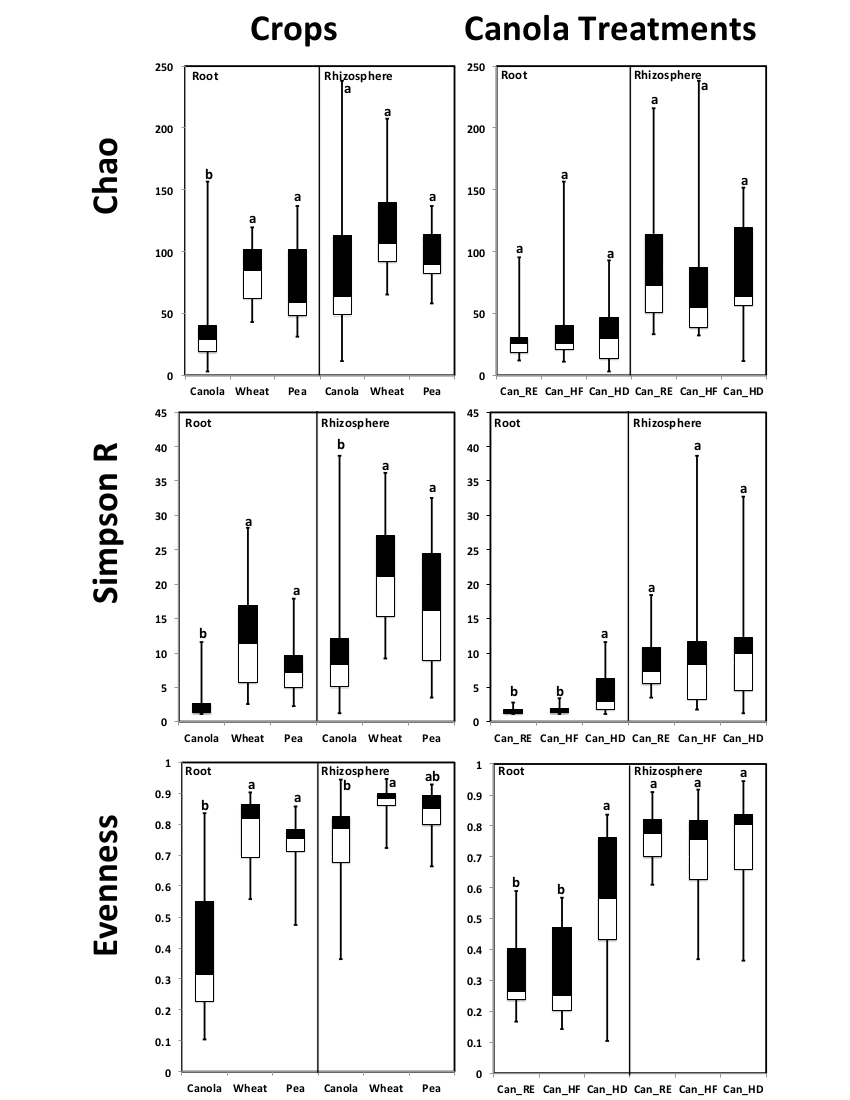

Supplement: Figure S4 — Biodiversity indices [Chao1, Simpson's reciprocal (Simpson R), and evenness] of fungal assemblages based on the operational taxonomic units (OTUs) of internal transcribed spacer (ITS) fragments. The figure shows the comparisons between crops (left) and canola treatments (right). The canola treatments were canola grown as recommended (Can_RE), canola fertilized at 150% of the recommended rate (Can_HF), and canola seeded at 150% of the recommended rate (Can_HD). The black and white boxes represent the median to the upper quartile and the median to the lower quartile, respectively. [file Image_4.TIFF]

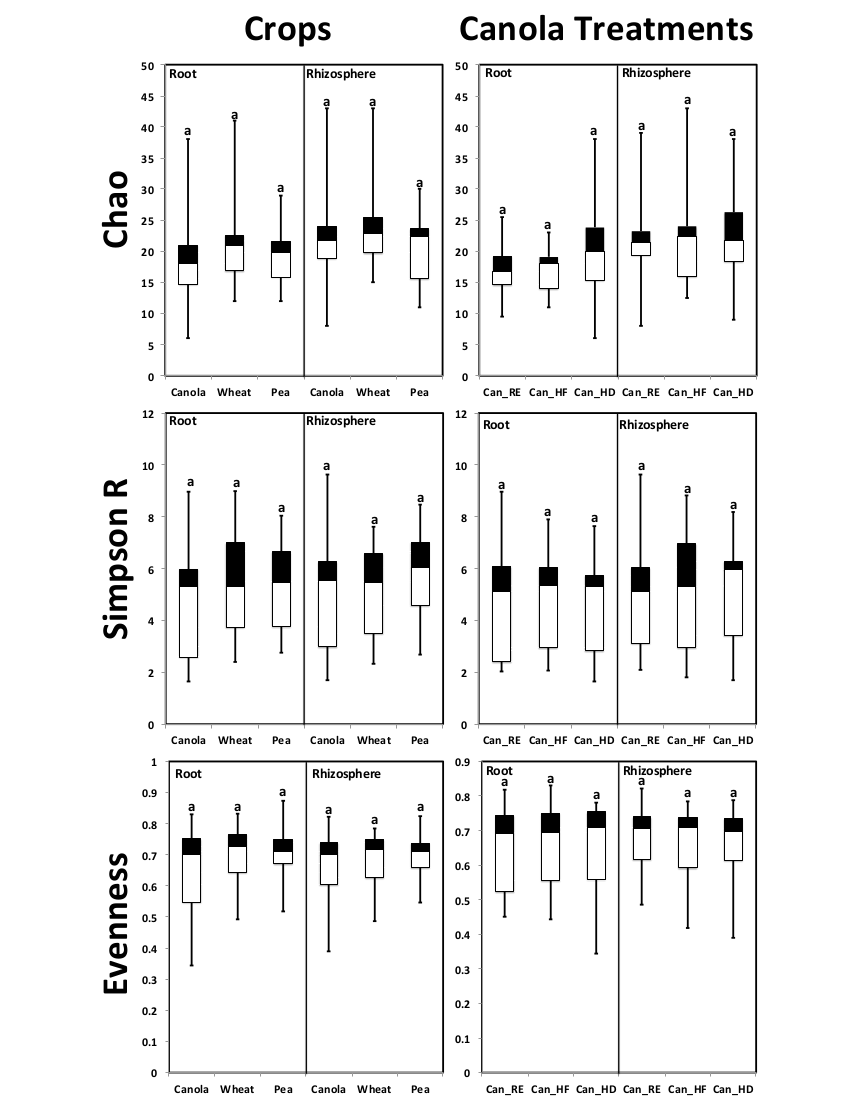

Supplement: Figure S5 — Biodiversity indices [Chao1, Simpson's reciprocal (Simpson R), and evenness] of archaeal assemblages based on the operational taxonomic units (OTUs) of 16S rRNA fragments. The figure shows the comparisons between crops (left) and canola treatments (right). The canola treatments were canola grown as recommended (Can_RE), canola fertilized at 150% of the recommended rate (Can_HF), and canola seeded at 150% of the recommended rate (Can_HD). The black and white boxes represent the median to the upper quartile and the median to the lower quartile, respectively. [file Image_5.TIFF]

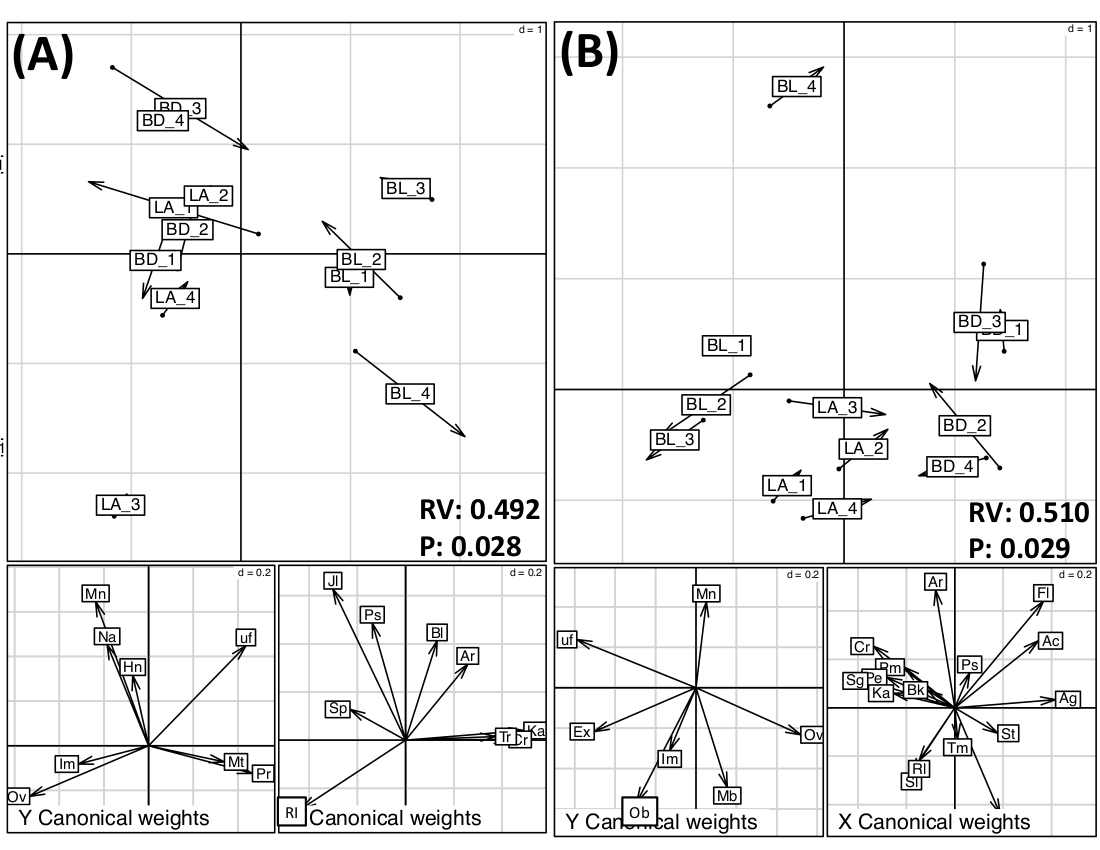

Supplement: Figure S6 — Co-inertia analysis showing the relationship between bacterial and fungal core and eco microbiomes in (A) the rhizosphere of pea and (B) the roots of wheat. For each crop, the top graph is the projection of both bacterial and fungal operational taxonomic units (OTUs) onto the co-inertia plane. The cumulative projective inertias for the first two axes were (A) 95.21% and (B) 89.41% of the total variation. The label abbreviations are as follows: BL, Beaverlodge; BD, Brandon; LA, Lacombe. Arrow length is proportional to the difference between the ordinations of the two data sets: longer arrows denote less concordance between the two assemblage data sets. The two sub-panels are the projection of fungi (left sub-panel) and bacteria (right sub-panel) onto the co-inertia plane for each crop. The abbreviations for bacterial and fungal species are as follows: Ac, Acidovorax radius; Ar, Arthrobacter sp.; Ag, Agrobacterium sp.; Bl, Blastococcus sp.; Bk, Burkholderia sp.; Cr, Cryococcus sp.; Fl, Flavobacterium sp.; Jl, Janthinobacterium lividum; Ka, Kaistobacter sp.; Pe, Pedobacter sp.; Pm, Promicromonospora sp.; Ps, Pseudomonas sp.; Rl, Rhizobium leguminosarum; Sg, Sphingomonas sp.; Sl, Salinibacterium sp.; Sn, Stenotrophomonas sp.; Sp, Serratia proteamaculans; St, Streptomyces sp.; Tm, TM7-3; Tr, Terracoccus sp.; Ex, Exophiala equine; Hn, Humicola nigrescens; Im, Ilyonectria macrodidyma; Ob, Olpidium brassicae; Ov, Olpidium virulentus; Mb, Microdochium bolleyi; Mn, Monographella cucumerina; Mt, Mortierella sp.; Na, Nectria ramulariae; Pr, Pseudogymnoascus roseus; uf, unclassified fungus. The RV coefficients are the values of the multivariate generalization of the squared Pearson correlation coefficient. P indicates the P-values of each analysis. [file Image_6.TIFF]

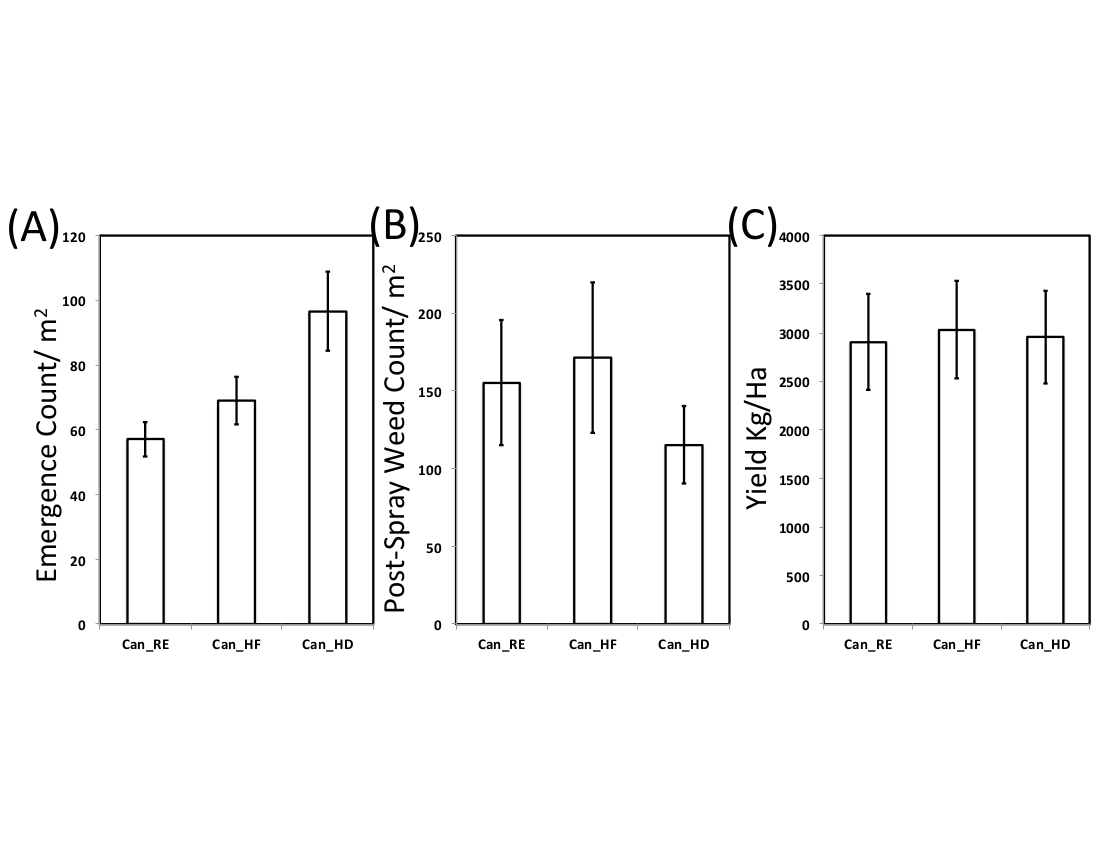

Supplement: Figure S7 — Agronomic variables of (A) canola emergence counts, (B) post-spray weed count, and (C) canola yield. The canola treatments were canola grown as recommended (Can_RE), canola fertilized at 150% of the recommended rate (Can_HF), and canola seeded at 150% of the recommended rate (Can_HD). [file Image_7.TIFF]
