# Supplementary material for: Canola Root–Associated Microbiomes in the Canadian Prairies
Source: Front Microbiol. 2018 Jun 8;9:1188. doi: 10.3389/fmicb.2018.01188 (PMC6002653; doi:10.3389/fmicb.2018.01188)
Supplement: Table S1 — Soil properties at each location. [file Table_1.DOCX]

**TABLE S1 |** Soil properties at each location.

| Location | pH | OM^a^  (g kg^−1^ ) | EC^b^  (ds m^−1^) | 0–15 cm  (mg kg^−1^) | | | | 15–60 cm (mg kg^−1^) | | Sand (%) | Silt (%) | Clay (%) | Texture | Group |
| --- | --- | --- | --- | --- | --- | --- | --- | --- | --- | --- | --- | --- | --- | --- |
|  |  |  |  | **N** | **P** | **K** | **S** | **N** | **S** |  |  |  |  |  |
| Lacombe, Alberta | 7.1–7.6 | 83 | 1.01- 1.44 | 11–15 | 19–24 | 144–154 | 111–466 | 3–4 | 170–766 | 46 | 33 | 21 | Loam | Penhold Black Chernozem |
| Beaverlodge, Alberta | 5.6–5.9 | 73 | 0.32–0.44 | 11–14 | 26–36 | 403–426 | 26–34 | 2–4 | 9–15 | 32 | 47 | 21 | Silt loam | Albright Gleyed Dark Gray Luvisol |
| Brandon, Manitoba | 7.8 | 53 | 0.54 | 10–14 | 5–6 | 190–254 | 8–21 | 27–47 | 26–60 | 42 | 28 | 30 | Clay loam | Newdale Orthic Black Chernozem |

^a^OM, organic matter.

^b^EC, electrical conductivity.

**TABLE S2 |** **The raw sequencing counts of each sample for bacteria, fungi and archaea by MiSeq Sequencing**

This table is an Excel file available in the file ‘TABLE S2.xls’

**TABLE S3 |** Effects of crop identity and canola treatments on the Chao1, Simpson’s reciprocal (Simpson R), and evenness indices, as determined by analysis of variance (ANOVA). Significant effects greater than *P* < 0.05 are indicated in bold.

| **Comparisons between crops** | | | | | | | |
| --- | --- | --- | --- | --- | --- | --- | --- |
|  | **Index** | **Biotope** | | **Crop** | | **Biotope*Crop** | |
|  |  | ***F*** | ***P*** | ***F*** | ***P*** | ***F*** | ***P*** |
| Bacteria | Chao1 | 72.26 | **<0.001** | 13.89 | **<0.001** | 10.15 | **<0.001** |
|  | Simpson R | 54.418 | **<0.001** | 6.755 | **0.002** | 1.262 | 0.287 |
|  | Evenness | 189.5 | **<0.001** | 125.2 | **<0.001** | 119.8 | **<0.001** |
| Fungi | Chao1 | 31.058 | **<0.001** | 9 | **<0.001** | 1.12 | 0.330 |
|  | Simpson R | 39.45 | **<0.001** | 25.79 | **<0.001** | 0.22 | 0.803 |
|  | Evenness | 75.455 | **<0.001** | 36.027 | **<0.001** | 9.031 | **<0.001** |
| Archaea | Chao1 | 7.488 | **0.007** | 1.542 | 0.218 | 0.391 | 0.678 |
|  | Simpson R | 0.256 | 0.614 | 1.122 | 0.329 | 0.119 | 0.888 |
|  | Evenness | 0.001 | 0.98 | 1.167 | 0.315 | 0.099 | 0.906 |
| **Comparisons between canola treatments** | | | | | | | |
|  | **Index** | **Biotope** | | **Treatment** | | **Biotope*Treatment** | |
|  |  | ***F*** | ***P*** | ***F*** | ***P*** | ***F*** | ***P*** |
| Bacteria | Chao | 19.155 | **<0.001** | 0.005 | 0.995 | 0.03 | 0.971 |
|  | Simpson R | 18.225 | **<0.001** | 0.058 | 0.944 | 0.096 | 0.909 |
|  | Evenness | 22.365 | **<0.001** | 0.224 | 0.8 | 0.201 | 0.818 |
| Fungi | Chao | 20.666 | **<0.001** | 0.076 | 0.927 | 0.215 | 0.807 |
|  | Simpson R | 26.649 | **<0.001** | 0.931 | 0.399 | 0.178 | 0.837 |
|  | Evenness | 72.112 | **<0.001** | 3.494 | **0.036** | 4 | 0.329 |
| Archaea | Chao | 6.239 | **0.015** | 0.248 | 0.781 | 0.281 | 0.756 |
|  | Simpson R | 0.297 | 0.587 | 0.036 | 0.965 | 0.015 | 0.985 |
|  | Evenness | 0.071 | 0.791 | 0.06 | 0.942 | 0.089 | 0.915 |

**TABLE S4 |** Canola bacterial and archaeal core and eco microbiomes among different treatments.

|  | | | **Biotope** | | | | | | | | | | | | |  |
| --- | --- | --- | --- | --- | --- | --- | --- | --- | --- | --- | --- | --- | --- | --- | --- | --- |
|  |  |  | **Root** | | | | | | **Rhizosphere** | | | | | | |  |
| **Taxonomy (phylum/class)^a^** | **Genus/species/**  **other** | | **Can_RE^b^**  **(%)** | **Can_HF**  **(%)** | **Can_HD**  **(%)** | **Core/**  **eco^c^** | **FDR *P*^d^** | **Sig^e^** | **Can_RE**  **(%)** | **Can_HF**  **(%)** | **Can_HD**  **(%)** | | **Core/eco** | **FDR *P*** | **Sig** | **OTU^f^** |
| Actino | *Streptomyces* sp. | | 6.29 | 4.62 | 2.68 | C | 0.17 |  | -^g^ | - | | - |  | - | - | OTU-B1 |
|  | *Cryocola* sp. | | 2.82 | 1.66 | 4.26 | C | 0.71 |  | 1.44 | 1.15 | | 1.76 | C | 0.99 |  | OTU-B2 |
|  | *Arthrobacter* sp. | | 2.78 | 2.78 | 2.25 | C | 0.94 |  | 4.48 | 4.99 | | 3.62 | C | 0.72 |  | OTU-B3 |
|  | *Amycolatopsis* sp. | | 1.49 | 0.57 | 0 | E | <0.001 | ** | - | - | | - |  | - | - | OTU-B4 |
|  | *Terracoccus* sp. | | - | - | - |  | - | - | 1.08 | 1.03 | | 0.95 | C | 0.99 |  | OTU-B5 |
|  | *Blastococcus* sp.^h^^ | | - | - | - |  | - | - | 1.30 | 0.95 | | 1.27 | C | 0.15 |  | OTU-B6 |
| Bact | *Flavobacterium* sp. | | 3.93 | 3.60 | 3.02 | C | 0.85 |  | - | - | | - |  | - | - | OTU-B7 |
|  | *Pedobacter* sp. | | 1.12 | 1.04 | 1.00 | C | 0.94 |  | - | - | | - |  | - | - | OTU-B8 |
| α | *Kaistobacter* sp. | | 1.45 | 1.32 | 1.42 | C | 0.97 |  | 2.29 | 2.00 | | 2.36 | C | 0.99 |  | OTU-B9 |
|  | *Agrobacterium* sp. | | 1.2 | 1.02 | 1.19 | C | 0.94 |  | - | - | | - |  | - | - | OTU-B10 |
| β | *Janthinobacterium lividum* | | 2.18 | 2.05 | 1.68 | C | 0.88 |  | 1.08 | 0 | | 0 | E | <0.001 | *** | OTU-B11 |
|  | *Burkholderia* sp. ^^^ | | 1.47 | 0.84 | 1.04 | C | 0.22 |  | - | - | | - |  | - | - | OTU-B12 |
|  | *Acidovorax radices* ^^^ | | 1.45 | 1.98 | 1.38 | C | 0.97 |  | - | - | | - |  | - | - | OTU-B13 |
| γ | *Serratia proteamaculans* ^^^ | | 7.20 | 10.10 | 11.46 | C | 0.94 |  | 9.60 | 8.47 | | 0 | E | <0.001 | ** | OTU-B14 |
|  | *Pseudomonas* sp. | | 4.32 | 6.04 | 4.81 | C | 0.88 |  | 1.90 | 2.40 | | 1.58 | C | 0.96 |  | OTU-B15 |
|  | *Erwinia* sp. | | 1.49 | 2.52 | 1.36 | C | 0.17 |  | - | - | | - |  | - | - | OTU-B16 |
|  | *Stenotrophomonas* sp. | | 0 | 1.04 | 1.44 | E | <0.001 | ** | 0.59 | 0.79 | | 1.2 | C | 0.89 |  | OTU-B17 |
|  | *Stenotrophomonas rhizophila* ^^^ | | 0.73 | 1.86 | 0.96 | C | 0.94 |  | - | - | | - |  | - | - | OTU-B18 |
|  | *Enterobacter* sp. ^^^ | | 0 | 2.56 | 0 | E | <0.001 | *** | - | - | | - |  | - | - | OTU-B19 |
| Sørensen index^i^ | | |  | 0.22  ± 0.0017 | 0.18  ± 0.0021 |  |  | *** |  | 0.32  ± 0.0012 | | 0.24  ± 0.0011 |  |  | *** |  |
| Thaumarchaeota | | OTU-A2 | - | - | - |  | - | - | 0 | 2.13 | | 0 | E | <0.001 | *** |  |
|  |  | OTU-A3 | 7.14 | 7.32 | 0 | E | <0.001 | *** | 0 | 10.18 | | 0 | E | <0.001 | *** |  |
|  |  | OTU-A4 | 26.90 | 24.42 | 24.19 | C | 1 |  | 25.58 | 26.78 | | 23.96 | C | 1 |  |  |
| Sørensen index | | |  | 0.05  ± 0.01 | 0.31  ± 0.007 |  |  | *** |  | 0.45  ± 0.01 | | 0.00  ± 0.00 |  |  | *** |  |

^a^Actino, *Actinobacteria*; Bact, *Bacteroidetes*; α, *Alphaproteobacteria*; β, *Betaproteobacteria*; γ, *Gammaproteobacteria*.

^b^Treatments were canola grown as recommended (Can_RE), canola fertilized at 150% of the recommended rate (Can_HF), and canola seeded at 150% of the recommended rate (Can_HD)

^c^C, core microbiome; E, eco microbiome.

^d^FDR *P*, false-discovery-rate-corrected *P*‑value.

^e^Sig, significance level: **P* ˂ 0.05; ***P* ˂ 0.01; ****P* ˂ 0.001.

^f^OTU, operational taxonomic unit.

^g^The symbol “-” indicates that the OTU does not meet the criteria for the core/eco microbiome.

^h^When followed by the symbol “^”, the classification was done by BLASTn manually with the NCBI database.

^i^The Sørensen index compares the assemblages between Can_HF and Can_HD with Can_RE.

**TABLE S5 |** Canola fungal core and eco microbiomes among different treatments.

|  | | **Biotope** | | | | | | | | | | | |  |
| --- | --- | --- | --- | --- | --- | --- | --- | --- | --- | --- | --- | --- | --- | --- |
|  |  | **Root** | | | | | | **Rhizosphere** | | | | | |  |
| **Taxonomy (phylum)** | **Genus/species** | **Can_ RE**  **(%)^a^** | **Can_ HF**  **(%)** | **Can_HD**  **(%)** | **Core/**  **eco^b^** | **FDR *P*^c^** | **Sig^d^** | **Can_ RE**  **(%)** | **Can_ HF**  **(%)** | **Can_HD**  **(%)** | **Core/**  **eco** | **FDR *P*** | **Sig** | **OTU^e^** |
| Ascomycota | *Fusicolla* sp.^f^^ | -^g^ | - | - |  | - | - | 5.08 | 4.40 | 0 | E | <0.001 | *** | OTU-F1 |
|  | *Fusarium merismoides* ^^^ | - | - | - |  | - | - | 6.10 | 7.11 | 8.27 | C | 0.89 |  | OTU-F2 |
|  | *Candida* sp. | 2.17 | 0 | 0 | E | <0.001 | *** | - | - | - |  | - | - | OTU-F3 |
|  | *Nectria ramulariae* | - | - | - |  | - | - | 1.29 | 0 | 0 | E | <0.001 | *** | OTU-F4 |
|  | *Humicola nigrescens* | - | - | - |  | - | - | 0 | 0 | 1.15 | E | <0.001 | *** | OTU-F5 |
|  | *Solicoccozyma aeria* | - | - | - |  | - | - | 0 | 0 | 1.22 | E | <0.001 | *** | OTU-F6 |
|  | *Mortierella* sp. | - | - | - |  | - | - | 0 | 1.56 | 0 | E | <0.001 | *** | OTU-F7 |
|  | *Monographella cucumerina* | - | - | - |  | - | - | 2.03 | 0 | 0 | E | <0.001 | ** | OTU-F8 |
| Chytridiomycota | *Olpidium brassicae* | 83.13 | 77.78 | 48.51 | C | <0.001 | * | 23.71 | 29.47 | 25.27 | C | 0.89 |  | OTU-F9 |
| Unclassified | Unidentified soil fungus | - | - | - |  | - | - | 1.76 | 2.85 | 2.37 | C | 0.89 |  | OTU-F10 |
| Sørensen index^h^ | |  | 0.25  ± 0.012 | 0.25  ± 0.012 |  |  |  |  | 0.32  ± 0.009 | 0.48  ± 0.008 |  |  | *** |  |

^a^Treatments were canola grown as recommended (Can_RE), canola fertilized at 150% of the recommended rate (Can_HF), and canola seeded at 150% of the recommended rate (Can_HD).

^b^C, core microbiome; E, eco microbiome.

^c^FDR *P*, false-discover-rate-corrected *P*‑value.

^d^Sig, significance level: **P* ˂ 0.05; ***P* ˂ 0.01; ****P* ˂ 0.001.

^e^OTU, operational taxonomic unit.

^f^When followed by the symbol “^”, the classification was done by BLASTn manually with the NCBI database.

^g^The symbol “-” indicates that the OTU does not meet the criteria for the core/eco microbiome.

^h^The Sørensen index compares the assemblages between Can_HF and Can_HD with Can_RE.

**TABLE S6 |** Significance of the relationships between the bacterial and fungal core/eco microbiomes, as determined by co-inertia analysis.

| **Biotope^a^** | **Treatment^b^** | **Cumulative projective inertia for the first two axes (%)** | **RV** | ***P*-value^c^** |
| --- | --- | --- | --- | --- |
| Roots | Can_RE | 100 | 0.201 | 0.224 |
|  | Can_HF | – | – | – |
|  | Can_HD | – | – | – |
|  | **Wheat** | **89.41** | **0.510** | **0.026** |
|  | Pea | 97.66 | 0.220 | 0.393 |
| Rhizosphere | **Can_RE** | **92.58** | **0.605** | **0.002** |
|  | **Can_HF** | **96.49** | **0.456** | **0.045** |
|  | **Can_HD** | **95.88** | **0.491** | **0.017** |
|  | Wheat | 87.04 | 0.266 | 0.508 |
|  | **Pea** | **95.21** | **0.492** | **0.027** |

^a^Biotopes are the plant roots and the rhizosphere soil.

^b^Treatments are canola grown as recommended (Can_RE), canola fertilized at 150% of the recommended rate (Can_HF), canola seeded at 150% of the recommended rate (Can_HD), wheat, and pea.

^c^Bold numbers indicate the significant models (*P* < 0.05). The symbol “–” indicates that core/eco bacterial and fungal assemblages in canola roots under the Can_HF and Can_HD treatments were not compared, since the fungal core microbiome was formed of only one species, *Olpidium brassicae*, preventing the comparison in the fungal core microbiome.

**TABLE S7 |** Comparison of the bacterial core microbiome of canola from this study with bacterial taxa considered important in other studies.

| **Phylum^a^** | **Genus** | **Germida et al., 1998** | **Macrae et al., 2000** | **Alström, 2001** | **Misko and Germida, 2002** | **Farina et al., 2012** | **Croes et al., 2013** | **de Campos et al., 2013** | **Tkacz et al., 2015** | **This study** | **PGPR^b^** |
| --- | --- | --- | --- | --- | --- | --- | --- | --- | --- | --- | --- |
| Location | | Canada | Britain | Sweden | Canada | Brazil | Belgium | Brazil | Britain | Canada |  |
| Method^c^ | | cd | ci | cd | cd | cd | cd | ci | ci | ci |  |
| Canola species and variety^d^ | | *B. napus* (Westar) | *B. napus* | *B. napus* (Casino) | *B. napus*^e^ | *B. napus* (Hyola 60) | *B. napus* | *B. napus* (Hyola 60) | *B. rapa* (R-O-18) | *B. napus* (InVigor L135C) |  |
| Actin | *Streptomyces* sp. |  |  |  |  | rt (1) ^f^ | rz |  |  | rt | yes in Pliego et al., 2011 |
|  | *Cryocola* sp. |  |  |  |  |  |  |  |  | rt/rz | no |
|  | *Arthrobacter* sp. | rt |  |  | rt/rz |  | rz |  |  | rt/rz | yes in Kloepper et al., 1988 |
|  | *Amycolatopsis* sp. |  |  |  |  |  |  |  |  | rt | no |
|  | *Blastococcus* sp. |  |  |  |  |  |  |  |  | rz | yes in Poly et al., 2001 |
|  | *Terracoccus* sp. |  |  |  |  |  |  |  |  | rz | no |
| Bact | *Flavobacterium* sp. | rt |  |  | rt/rz |  |  | rt (fl) |  | rt | yes in Belimov and Dietz, 2000 |
|  | *Pedobacter* sp. |  |  |  |  | rt/rz (4) | rt | rt (fl) | rz | rt | no |
| α | *Kaistobacter* sp. |  |  |  |  |  |  |  |  | rt/rz | no |
|  | *Agrobacterium* sp. |  |  |  |  | rt/rz (4) | rz |  |  | rt | no |
| β | *Janthinobacterium* sp. |  |  |  |  |  | rz (genus) |  |  | rt | yes in Cruz et al., 2008 |
|  | *Burkholderia* sp. |  |  |  |  | rt/rz (4) |  |  |  | rt | yes in Gray and Smith, 2005 |
|  | *Acidovorax* sp. |  |  |  |  |  |  |  |  | rt | no |
| γ | *Serratia* sp. |  |  | rt |  | rt/rz (3) |  |  |  | rt/rz | yes in Alström, 2001 |
|  | *Pseudomonas* sp. |  |  | rt | rt/rz | rt/rz (4) | rt/rz | rt (ro/fl) |  | rt/rz | yes in Gray and Smith, 2005 |
|  | *Erwinia* sp. |  |  |  |  |  |  |  |  | rt | no |
|  | *Acinetobacter* sp. |  |  |  |  | rz (1) |  |  |  | rt | no |
|  | *Stenotrophomonas* sp. |  |  | rt | rt/rz | rt/rz (3) | rt | rt(fl) |  | rt | yes in Alström, 2001 |
|  | *Enterobacter* sp. |  |  | rt |  | rt/rz (4) |  | rt |  | rt/rz | yes in Alström, 2001 |
| Fir | *Bacillus* sp. | rt | rz |  |  |  | rt/rz |  |  |  | yes in Kumar et al., 2011 |
|  | *Staphylococcus* sp. | rt |  |  |  |  |  |  |  |  | no |
|  | *Oxalophagus* sp. |  |  |  |  |  |  |  | rz |  | no |
| Actin | *Curtobacterium* sp. | rt |  |  |  |  |  |  |  |  | no |
|  | *Micrococcus* sp. | rt |  |  |  |  |  |  |  |  | no |
|  | *Rathayibacter* sp. | rt |  |  |  |  |  |  |  |  | no |
| Bact | *Chryseobacterium* sp. |  |  |  |  |  |  | rt (ro/fl) |  |  | no |
|  | *Sphingobacterium* sp. |  |  |  |  |  |  | rt (ro/fl) |  |  | no |
| α | *Asticcacaulis* sp. |  |  |  |  |  |  |  | rz |  | no |
|  | *Caulobacter* sp. |  |  |  |  |  | rt/rz |  | rz |  | no |
|  | *Labrys* sp. |  |  |  |  |  | rt/rz |  |  |  | no |
|  | *Rhizobium* sp. |  |  |  |  | rt/rz (3) |  | rt (fl) | rz |  | no |
|  | *Mesorhizobium* sp. |  |  |  |  |  |  |  | rz |  | no |
| β | *Alcaligenes* sp. |  |  | rt |  | rt (2) |  |  |  |  | no |
|  | *Variovorax* sp. |  |  |  |  |  | rt/rz |  |  |  | no |
|  | *Achromobacter* sp. |  |  |  |  | rz/rt (2) |  |  |  |  | no |
|  | *Massilia* sp. |  |  |  |  |  | rz | rt(ro/fl) | rz |  | no |
| γ | *Xanthomonas* sp. |  |  |  |  | rt/rz (2) |  | rt (fl) |  |  | no |
|  | *Klebsiella* sp. |  |  |  |  | rt/rz (2) |  |  |  |  | no |
|  | *Pantoea* sp. |  |  |  |  | rt/rz (4) |  |  |  |  | no |
|  | *Rhodanobacter* sp. |  |  |  |  |  |  |  | rz |  | no |
|  | *Solimonas* sp. |  |  |  |  |  |  |  | rz |  | no |
| TM7 | Unknown TM7 |  |  |  |  |  |  |  | rz |  | no |
| note |  | Only endophyte isolates | Low sequence identifica-tion |  | Focused on *Pseudo-monas* |  | Present in both rt and rz | Greater than 1% in the sample | Requested directly from the author | Core microbi-ome > 1% |  |

^a^Actin: *Actinobacteria*; Bact, *Bacteroidetes*; α, *Alphaproteobacteria*; β, *Betaproteobacteria*; γ, *Gammaproteobacteria*; Fir, *Firmicutes*.

^b^PGPR: reported as plant-growth-promoting rhizobacteria in the literature.

^c^cd: culture-dependent; ci, culture-independent.

^d^*B. napus*: *Brassica napus*; *B. rapa*, *Brassica rapa*.

^e^The cultivars used in the study by Misko (2002) were Exceed, Innovator, Quest RR, AC-excel, Fairview, and Hyola 401.

^f^rt: roots; rz: rhizosphere; fl: flowering stage; ro: rosette stage. In Farina (2012), numbers in brackets are the frequency of the genus detected in the fields.
