# Supplementary material for: Canola Root–Associated Microbiomes in the Canadian Prairies
Source: Front Microbiol. 2018 Jun 8;9:1188. doi: 10.3389/fmicb.2018.01188 (PMC6002653; doi:10.3389/fmicb.2018.01188)
Supplement: Supplementary file 10 [file Data_Sheet_1.DOCX]

Supplementary information

**Materials and Methods**

*Additionnal details on the field experiments*

The soils at the site were a Penhold Black Chernozem in Lacombe, AB (52.58N, 113.78W); an Albright Dark Gray Chernozem in Beaverlodge, AB (55.28N, 119.48W); and a Newdale Orthic Black Chernozem in Brandon, MB (50.08N, 99.98W) (Harker et al., 2015). The textures of the soils were loam, silt loam, and clay loam in Lacombe, Beaverlodge, and Brandon, respectively. Lacombe had the highest soil organic matter content at 8.3%, while Brandon had the lowest content at 5.3%. The soil in Beaverlodge was slightly acidic (pH = 5.6-5.9), while Lacombe (pH = 7.1–7.6) and Brandon (pH = 7.8) had slightly alkaline soils.

Each experimental field was arranged in a randomized complete block design with four blocks, and each received the same five treatments (Table 1). From 2008 to 2013, the fields were rotated with different canola cultivars or other rotation crops (wheat [*Triticum aestivum* L.] and pea [*Pisum sativum* L.]). In 2013, all fields were seeded with the canola cultivar 6056 CR (BrettYoung, Leduc, AB), a glyphosate-resistant canola cultivar. In 2014, the year of sampling, the plots of the first three practices (Can_RE, Can_HF, and Can_HD) were planted with the canola cultivar InVigor L135C (Bayer Crop Science, Calgary, AB), a clubroot-resistant canola cultivar that is resistant to the herbicide glufosinate.

Soil samples were collected at each site before seeding and analyzed for soil nutrients (Table 1). On the basis of the soil analyses, fertilizer additions (N, P_2_O_5_, K_2_O, S) were made to achieve 100% or 150% of the soil test recommendations for each crop species. Most fertilizer was side-banded 2 cm beside and 3 to 4 cm below the seed row with small amounts of nitrogen and phosphorus also placed with crop seeds (for details, see Harker et al. 2015). Nitrogen was added as ESN Smart Nitrogen (Agrium Inc.), phosphorus as diammonium phosphate, potassium as muriate of potash, and sulphur as elemental sulphur. Seeding was performed with an air seeder equipped with knife openers, and crops were seeded at about an 18-mm depth in rows 20- to 30-cm apart. Plot dimensions were 3.7×15.2 m. Herbicides were applied: canola plots received glufosinate at 500 g ai ha^-1^ + clethodim at 15 g ai ha^-1^ + phosphate ester surfactant at 0.5% v/v; pea plots received imazamox at 20 g ai ha^-1^ + quizalofop-p-ethyl at 36 g ai ha^-1^ + surfactant and petroleum hydrocarbon blend (Merge, BASF) at 0.5% v/v; for wheat, various herbicides were applied to wheat plots based on weed populations at each location.

*Additional variables*

In each plot, the emergence date was determined every 2–3 days. Crop emergence counts were assessed 2–3 weeks after emergence on two 1-m rows per plot. Weed counts were taken 3–4 weeks after herbicide application. All weeds were counted from all crops at the same time from pre-marked quadrats. Weed biomass was assessed at maturity from the same pre-marked quadrats. The crop maturity date was determined every 2–3 days and recorded in Julian days. The results are shown in Fig S7.

*Additionnal details on DNA isolation*

Total root DNA was extracted from 350 mg of root material using the NucleoSpin Soil DNA isolation kit (Macherey-Nagel, Düren, Germany) according to the manufacturer’s instructions with the following modifications: NucleoSpin® Bead tubes with Lysis buffer L1 were placed in a FastPrep^TM^ FP120 Homogenizer (ThermoFisher Scientific, Waltham, MA) and bead beating was used to further break down root material, using a speed of 5 m s^-1^ for 3×20 s with 30-s gaps in between each repeat.

Microbial genomic DNA from rhizosphere soil was isolated using the RNA PowerSoil Total RNA Isolation Kit plus the RNA PowerSoil DNA Elution Accessory Kit (Mo Bio Laboratories Inc., Carlsbad, CA, USA). Samples were prepared according to the manufacturer’s instructions with some modifications: 1) the bead-beating step was extended to 20 min, 2) the first precipitation at -20°C was ~30 min, 3) sample tubes were kept on ice as much as possible, and 4) the second precipitation was not shorter than 30 min at -20°C.

*Details on amplicon library preparation and sequencing*

The PCR reaction conditions were as follows: 95°C for 3 min, 40 cycles of 98°C for 20 s, 55°C (for bacteria) / 60°C (for archaea) / 45°C (for fungi) for 30 s, and 72°C for 45 s, with a final extension at 72°C for 7 min. PCR products were verified on 1% agarose gels using electrophoresis.

Dual Nextera indices were then attached to PCR products based on the suggested protocol “16S Metagenomic Sequencing Library Preparation” provided by Illumina with certain modifications, including 1) the reaction volume was reduced from 50 μL to 25 μL, 2) the first temperature for the 8-cycle PCR reaction for duplication was changed from 95°C to 98°C based on the manual for the KAPA HiFi HotStart ReadyMix, and 3) AMPure XP beads were replaced with the NucleoMag® NGS Clean-Up and Size Select kit (Macherey-Nagel), which was used according to the manual provided by the manufacturer. The final purified product was 27.5 μL per reaction, which was quantified by Qubit Fluorometric Quantitation (ThermoFisher Scientific). The Amplicon pool was constructed by adding 15 ng of DNA from each library to the pool. The required amount of each sample was calculated based on the Qubit measurements. Bacterial, archaeal, and fungal libraries were pooled in equimolar ratios before sequencing into three distinct sample tubes. The pooled samples were concentrated down to about 30 μL using an Amicon 10K device (EMD, Millipore, Billerica, MA).

*Additionnal details on bioinformatics*

The function “make.contigs” was used to join the paired ends and “trim.seqs” was used with the options pdiff=2 and maxambig=0 to trim the sequences. We then used “list.seqs” and “get.seqs” to parse the sequences after trimming within each sample group. To reduce the file size for bacterial 16S rRNA gene sequences in order to accommodate the size limit of the 32-bit version of USEARCH (Edgar, 2010), we discarded singletons using “unique.seqs” and “split.abund” with a cutoff=1. This step was not required for the archaeal and fungal sequences, as the total file sizes were small. The files were then processed following the bacterial 16S pipeline (Pylro et al., 2014) using QIIME (Caporaso et al., 2010), USEARCH, the Fasta formatter (from the FASTX-toolkit; http://hannonlab.cshl.edu/fastx_toolkit/index.html), and scripts written by members of the BMP. Since the archaeal OTUs were not well identified, as the database for archaea was incomplete, we constructed an archaeal phylogenetic tree based on the OTUs of the members of the archaeal core microbiomes, which we had computed previously using the script “compute_core_microbiome.py” using phylogeny.fr (Dereeper et al., 2008). This program is an automatic pipeline for building phylogenetic trees from raw sequence files. Within this pipeline, MUSCLE (v3.7) was used to align the sequences, which were subsequently submitted to the curation program gBlocks® (v0.91b) to trim the ambiguous regions. The phylogenetic tree was built by PhyML using the maximum likelihood method with the HKY85 substitution model with 175 positions. Graphical representation and editing were performed by TreeDyn (v198.3). The phylogenetic tree improved the identification of OTUs with the reference sequences selected from GenBank.

*Additionnal details on statistical analysis*

To compare the clustering of samples, β-diversity was revealed by sample clustering in PCoA plots drawn using weighted UniFrac dissimilarity matrices for bacterial and archaeal OTUs using the QIIME script “core_diversity_analyses.py”, while a Bray-Curtis dissimilarity matrix was used for fungal OTUs. Figures were plotted using the R package “vegan”.

We considered the members of the “core microbiome” to be the microbes more influential and more prevalently distributed with the plant hosts. To achieve this goal, we first selected the principal microbial OTUs of each treatments. For bacterial and archaeal assemblages, we set the threshold for OTU prevalence across samples at 90%, i.e. we only retained the OTUs found in at least 11 of 12 samples (4 replicates in three locations) as candidates for core or eco microbiomes. That means that we allow the principal OTUs to miss in 1 of the 12 samples in each treatment. For fungal assemblages, we set the threshold at 75% (9 out of 12 samples) due to higher variability in fungal OTU distribution. The difference in the cut-off for bacteria and fungi is due to the evenness of OTU distributions—bacteria and archaea were more evenly distributed than fungi, and the 9 out of 12 samples for fungi are based on covering at least one replicate at one location for certain OTUs to be considered as core/eco microbiomes. That means we allow the principal fungal OTUs to be missing in 3 out of 12 samples to take into consideration. Only the OTUs that constituted the average proportions more than 1% of the assemblage for at least one of the combinations of crop or treatment were retained for further consideration, as we assumed that these were more influential to the host plants than the ones with less than 1% of the assemblage.

We used the genus/species names according to the databases for the OTUs. However, when the OTUs were not classified at least to the genus, we ran a BLASTn (Altschul et al., 1990) search against the NCBI database to obtain the closest classification (marked with the sign ^ in Tables 3–5). Kruskal-Wallis tests and Benjamini & Hochberg FDR P-values (Goeman and Solari, 2014) were used to evaluate the significance of the effect of crops and treatments.

**References**

Altschul, S.F., Gish, W., Miller, W., Myers, E.W., and Lipman, D.J. (1990). Basic Local Alignment Search Tool. *J Mol Biol* 215**,** 403–410.

Caporaso, J.G., Kuczynski, J., Stombaugh, J., Bittinger, K., Bushman, F.D., Costello, E.K., Fierer, N., Pena, A.G., Goodrich, J.K., Gordon, J.I., Huttley, G.A., Kelley, S.T., Knights, D., Koenig, J.E., Ley, R.E., Lozupone, C.A., Mcdonald, D., Muegge, B.D., Pirrung, M., Reeder, J., Sevinsky, J.R., Tumbaugh, P.J., Walters, W.A., Widmann, J., Yatsunenko, T., Zaneveld, J., and Knight, R. (2010). QIIME allows analysis of high-throughput community sequencing data. *Nat Methods* 7**,** 335–336.

Dereeper, A., Guignon, V., Blanc, G., Audic, S., Buffet, S., Chevenet, F., Dufayard, J.F., Guindon, S., Lefort, V., Lescot, M., Claverie, J.M., and Gascuel, O. (2008). Phylogeny.fr: robust phylogenetic analysis for the non-specialist. *Nucleic Acids Res* 36**,** W465–W469.

Edgar, R.C. (2010). Search and clustering orders of magnitude faster than BLAST. *Bioinformatics* 26**,** 2460–2461.

Goeman, J.J., and Solari, A. (2014). Multiple hypothesis testing in genomics. *Stat Med* 33**,** 1946–1978.

Harker, K.N., O'Donovan, J.T., Turkington, T.K., Blackshaw, R.E., Lupwayi, N.Z., Smith, E.G., Dosdall, L.M., Hall, L.M., Kutcher, H.R., Willenborg, C.J., Peng, G., Irvine, R.B., and Mohr, R. (2015). Canola cultivar mixtures and rotations do not mitigate the negative impacts of continuous canola. Can J Plant Sci 95, 1085-1099.

Pylro, V.S., Roesch, L.F.W., Morais, D.K., Clark, I.M., Hirsch, P.R., and Totola, M.R. (2014). Data analysis for 16S microbial profiling from different benchtop sequencing platforms. *J Microbiol Meth* 107**,** 30–37.
